# A Systematic Review and Network Meta-Analysis about the Efficacy and Safety of *Tripterygium wilfordii* Hook F in Rheumatoid Arthritis

**DOI:** 10.1155/2022/3181427

**Published:** 2022-05-10

**Authors:** Hai-long Wang, Qi Zhao, Wei Li, Hua-chao Zhu, Liu Lv, Zhen-hong Zhu, Xi-xi Wang, Zheng-zheng Yang, Yu-cao Ma, Ming-xuan Liu, Yi-wen Wang, Hezheng Lai, Chun-ping Liu, Yu-zheng Yang

**Affiliations:** ^1^Dongzhimen Hospital, Beijing University of Chinese Medicine, Beijing 100700, China; ^2^Chinese Medicine Centre, Western Sydney University, Penrith, NSW 2751, Australia; ^3^Guizhou University of Traditional Chinese Medicine, Guiyang 550025, China; ^4^Guang'anmen Hospital, China Academy of Chinese Medical Sciences, Beijing 100053, China; ^5^Beijing University of Chinese Medicine, Beijing 100700, China; ^6^The Affiliated Hospital of Guizhou Medical University, Guiyang 550001, China

## Abstract

**Objective:**

This study aims to evaluate the efficacy of various conventional synthetic DMARDs, including *Tripterygium wilfordii* Hook F (TwHF) for treating rheumatoid arthritis (RA) by network meta-analysis.

**Methods:**

We retrieved the related literature from online databases and supplemented it by using a manual retrieval method. Data was extracted from the literature and analyzed with STATA software.

**Results:**

A total of 21 trials (5,039 participants) were identified. Assessment of ACR20 response found that TwHF combined with methotrexate (MTX) had the greatest probability for being the best treatment option among the treatments involved, while TwHF used singly was second only to TwHF combined with MTX. Assessment of ACR50 response found that TwHF combined with MTX ranked second in all treatment options after cyclosporine A (CsA) combined with leflunomide (LEF) and TwHF alone, followed by TwHF combined with MTX. Assessment of ACR70 response found that CsA combined with LEF ranked first, TwHF combined with LEF ranked second, TwHF combined with MTX ranked third, and TwHF used singly ranked fourth. In the safety analysis, TwHF had the least probability of adverse event occurrence, followed by TwHF combined with MTX, which ranked first and second, respectively.

**Conclusion:**

Compared with the current csDMARDs for treating RA, the efficacy of TwHF was clear, and TwHF combined with MTX performed well under various endpoints. In the future, large, rigorous, and high-quality RCTs are still needed to confirm the benefits of TwHF therapy on RA.

## 1. Introduction

Rheumatoid arthritis (RA) is a common systemic immune disease which is characterized by joint inflammation, destruction, and deformity associated with chronicity and a high rate of disability. Improvement in treatment, stopping progression, and optimizing quality of life are priorities in the field of rheumatology in China. *Tripterygium wilfordii* Hook F (TwHF) refers to the dry root or root xylem of the celastraceae plant *Tripterygium wilfordii*, a widely used herb in traditional Chinese medicine (TCM). In accordance with TCM theory, TwHF is considered a key herb for treating persistent rheumatoid arthritis, due to its strong efficacy in eliminating wind-damp and promoting blood circulation to dredge collaterals. In recent years, TwHF prepared by extracting the essence of the active components of *Tripterygium wilfordii* has been used in clinical practice to treat a variety of rheumatic immune diseases, including RA [1–4]. It is noted that TwHF has been found to possess toxicity and is associated with having adverse events, such as hepatorenal toxicity, reproductive toxicity, and hematologic toxicity. Network meta-analysis (NMA) is a technique for comparing three or more interventions simultaneously in a single analysis by combining direct and indirect evidence and ranking the efficacy. Compared with traditional meta-analysis, NMA may assist in comparing the efficacy of multiple interventions for a disease more comprehensively, to provide more rigorous evidence through greater synthesis of information. There are many meta-analyses on TwHF in treating RA [5,6], but most are pairwise comparisons, which have limited ability to illustrate the individual differences among multiple disease-modifying antirheumatic drugs (DMARDs). This study is distinguished as a NMA which includes new and recent studies to evaluate the efficacy and safety of commonly used conventional synthetic DMARDs as both monotherapy and combination therapy for treatment of RA, including TwHF, methotrexate (MTX), leflunomide (LEF), sulfasalazine (SSZ), cyclosporine A (CsA), tacrolimus (FK506), minocycline (MINO).

## 2. Methods

### 2.1. Data Sources and Searches

This review was performed according to the Preferred Reporting Items for Systematic Review and Meta-Analysis (PRISMA) statement. We systematically searched the electronic databases PubMed, Embase, CNKI, Cochrane Library, SinoMed, and Wanfang Data from inception to February 28, 2020. We adopted a search method of subject words combined with free words, while manual retrieval was also performed to avoid omission. Searches included a combination of free text and Medline Subject Headings (MeSH) terms for “disease terms” with “drug names,” and were limited to published RCTs. For the English databases, we used free text terms, such as “*Tripterygium wilfordii* Hook F″, methotrexate, leflunomide, sulfasalazine, hydroxychloroquine, cyclosporine A, azathioprine, cyclophosphamide, mycophenolate mofetil, tacrolimus (FK506), intramuscular gold, auranofin, minocycline, D-penicillamine, chlorambucil, “rheumatoid arthritis”, and “randomized controlled trials”. For the Chinese databases, free texts were used, such as “Lei gong teng”, “Lei Gong Teng Zhiji”, “Lei Gong Teng Duo Gan”, “Jia An Die Ling (MTX)”, “Lai Fu Mi Te (LEF)”, “Liu Dan Huang Bi Ding (SSZ)”, “Huan Bao Su A (CsA)”, “Ta Ke Mo Si (FK506)”, “Mi Nuo Huan Su (MINO)”, “Lin Chuang Yan Jiu (clinical research)”,“Lei feng shi guan jie yan (rheumatoid arthritis)”, “Sui Ji Dui Zhao Shi Yan (RCT)”.

### 2.2. Study Selection

#### 2.2.1. Inclusion Criteria

Literature that met all the following requirements were included:

Types of studies:
  - Randomized controlled trials of conventional synthetic DMARDs for treatment of RA, published in either English or Chinese language.
Types of participants:
  - The subjects were diagnosed with RA in accordance with the 1987 Guidelines of the American Rheumatology Association [7] or the 2010 ACR/European League against Rheumatism (EULAR) Criteria [8]; without diagnosis of other autoimmune diseases or serious cardiovascular and cerebrovascular diseases; no restrictions on age, sex, race, or nationality.
Types of intervention:
  - TwHF, MTX, LEF, SSZ, CsA, FK506, and MINO used singly or as a two-drug combination in the treatment of RA. TwHF includes both tripterygium glycoside tablet and tripterygium tablet, the two root preparations of TwHF that have shown therapeutic promise [9,10]. The time limit for intervention was ≥12 weeks. Use of nonsteroidal anti-inflammatory drugs, folic acid, vitamins, calcium tablets, and low-dose hormones as adjuvant therapy during the treatment was not limited.
Types of outcome measures:
  - Primary outcome: the American College of Rheumatology (ACR) response criteria ACR20 [11].
  - Secondary outcomes: ACR50, ACR70, and incidence of adverse events. All literature studies on adverse events were included, inclusive of all types of adverse events;
  - The analyses of outcomes were conducted on an intent-to-treat (ITT) basis, or modified ITT (number actually receiving treatment at baseline) if the number randomized to treatment was not reported.

### 2.3. Exclusion Criteria

- Publications where full text literature cannot be obtained;
- Studies where research data are incomplete or cannot be extracted for analysis;
- Interventions as herbs containing TwHF.

### 2.4. Data Extraction and Quality Assessment

The literature screening and extraction were carried out by two researchers, respectively, according to the inclusion criteria for literature retrieval. After the preliminary screening of titles and abstracts, the full text was screened, and the literature inclusion and data extraction were carried out based on intentionality analysis. Finally, the data extracted was compared and sorted. Two authors independently evaluated the methodological quality of eligible publications by using the Cochrane Collaboration's tool for assessing the risk of bias [12] (random sequence generation, allocation concealment, blinding of participants and personnel, blinding of outcome assessment, incomplete outcome data, selective reporting, and other sources of bias). If there were differences, a third-party researcher was invited to assist the ruling.

### 2.5. Data Synthesis and Analysis

The primary outcome of this analysis was the American College of Rheumatology (ACR) response criteria: ACR20. The ACR20 is defined as a reduction by 20% or more, in the number of tender and swollen joints plus 20% improvement in at least three of the following five measures: pain, patient global assessment, physician global assessment, a score of physical disability, and blood acute-phase reactants. The secondary outcomes were ACR50, ACR70, and adverse events. The ACR50 is defined as an improvement of 50% or more in the number of tender and swollen joints, plus 50% improvement in at least three of the aforementioned five measures. The ACR70 is defined as an improvement of 70% or more in the number of tender and swollen joints, plus 70% improvement in at least three of the aforementioned five measures.

### 2.6. Network Meta-Analysis

Results are reported as odds ratios (ORs) with 95% confidence intervals (CI) for all comparisons of interventions. Initially, traditional pairwise meta-analysis was performed by using a random-effects model. Then network meta-analysis was performed to compare different therapies by using a frequentist approach. We included multi-arm trials in the analysis by breaking multi-arm trials into separate two-arm trials. We employed a multi-variate random-effects meta-analysis model for each outcome separately, combining direct evidence for each comparison [13,14].

For each “loop” of treatment comparisons from three or more independent sources and for each outcome, we computed the difference between estimates from direct and indirect evidence on the log OR scale. Inconsistency was defined as disagreement between direct and indirect evidence with a 95% CI excluding 0. For each outcome, we estimated the probability of which intervention was the best for each outcome, the second best, the third best, and so on, from the ranked order of the treatments at each interaction. These ranking probabilities were used to calculate the surface under the cumulative ranking curve (SUCRA), which is expressed as percentage (100% for the best intervention, 0% for the worst intervention, and approximately 50% for equivalent interventions) [15].

### 2.7. Funnel Plot and Publication Bias

The difference between the observed effect size and comparison-specific summary effect for each study was calculated. This variable was then regressed on the standard error (SE), thus adding a simple linear regression line in the funnel plot. This method could help to visually determine if there is a publication bias in the results between small and large studies. We performed traditional and network meta-analysis by using Stata software (version 12.0, the StataCorp, College Station, Texas, USA).

## 3. Results

**Figure 1:**
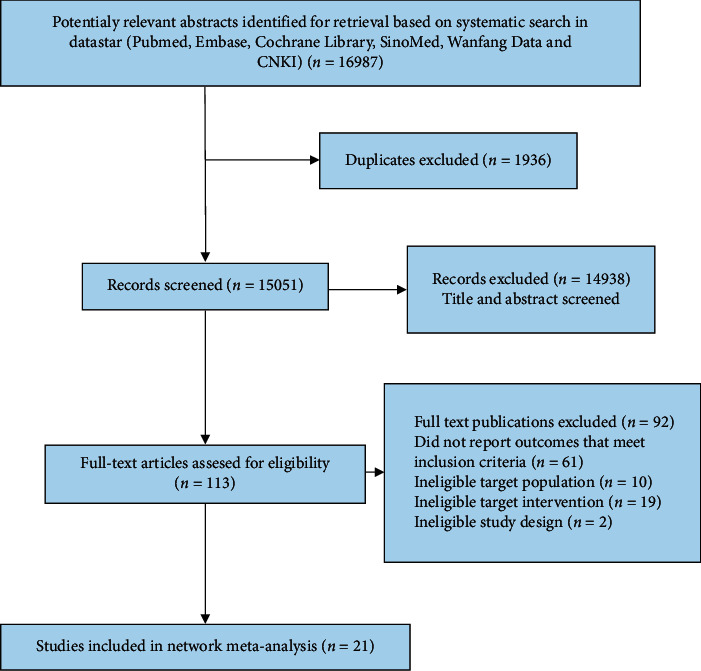
The flowchart.

**Figure 2:**
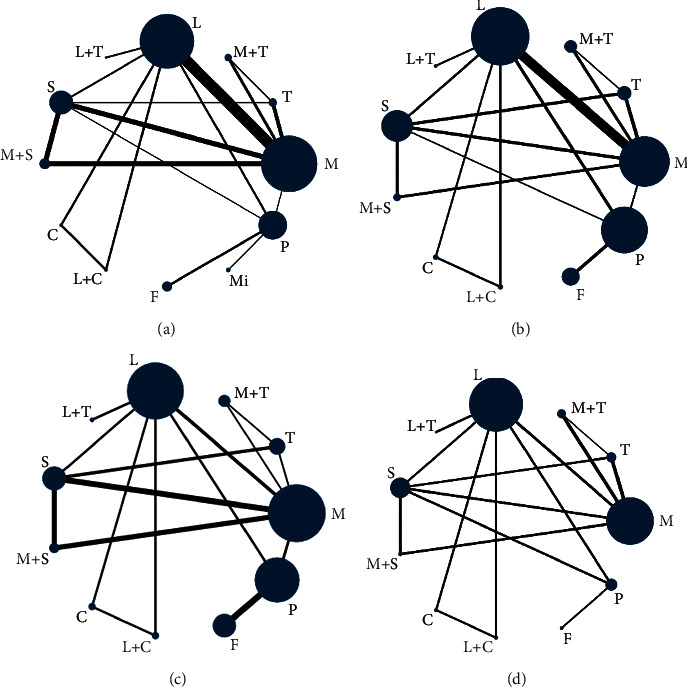
The network of all treatment comparisons analyzed according to ACR 20, 50, 70 response, and adverse events. (a) Network evidence plot based on ACR20. (b) Network evidence plot based on ACR50. (c) Network evidence plot based on ACR70 (d) and adverse events.

The flow chart of studies considered for inclusion is shown in Figure 1. On the basis of the title and abstract, 113 publications were selected and analyzed in full text versions. Eventually, 21 publications were included in the systematic review, and the characteristics of the literature were extracted as Table 1.  Figure 2 shows the network of all treatment comparisons analyzed according to ACR 20, 50, 70, and adverse events. All reviews followed the methods in the Cochrane Handbook, including standardized searches, inclusion criteria, and outcomes.

### 3.1. Characteristics of Included Studies

Table 1 summarizes the clinical and methodological characteristics as well as the main outcomes of each trial. A total of 21 trials (5,039 participants) were identified, and the characteristics of the literature were extracted as shown in Table 1. The risk of bias assessments for the included trials is illustrated in Figure S2 and Figure S3. Most of the evidence was of moderate-to-good quality. All 21 RCTs mentioned the word “randomization”. Over half of the studies did not report adequate information about allocation sequence generation and allocation sequence concealment. Unblinded designs were used in over half of the trials included.

### 3.2. NMA Results

#### 3.2.1. ACR20

In the evaluation of the ACR20 response, 21 studies were included, involving a total of 5039 patients, including a total of 12 kinds of interventions. The interventions were MTX, TwHF, TwHF combined with MTX, LEF, TwHF combined with LEF, SSZ, SSZ combined with MTX, CsA, CsA combined with LEF, FK506, MINO, and placebo (Figure S4A). Efficacy was evaluated by drawing cumulative probability diagram, probability efficacy ranking table (Table 2), and inverted triangle table (Table 3). According to the analysis results, TwHF combined with MTX had the greatest probability of the best efficacy among the treatments involved, and TwHF used singly ranked second (Figure S5A).

#### 3.2.2. ACR50

In the evaluation of ACR50 response, 15 literature studies were included, involving 2,968 patients, including 11 interventions: MTX, TwHF, TwHF combined with MTX, LEF, TwHF combined with LEF, SSZ combined with MTX, CsA, CsA combined with LEF, FK506, and placebo (Figure S4B). The efficacy was evaluated by drawing a cumulative probability diagram, a probability efficacy ranking table (Table 2), and an inverted triangle table (Table S1). According to the analysis results, the efficacy of TwHF combined with MTX ranked second only to CsA combined with LEF in all treatment schemes, while TwHF alone ranked third in all treatment schemes, second only to TwHF combined with MTX (Figure S5B).

#### 3.2.3. ACR70

In the evaluation of ACR70 response, 10 literature studies were included, involving 2,374 patients, including 11 interventions: MTX, TwHF, TwHF combined with MTX, LEF, TwHF combined with LEF, SSZ, SSZ combined with MTX, CsA, CsA combined with LEF, FK506 and placebo (Figure S4C). The efficacy was evaluated by drawing a cumulative probability diagram, a probability efficacy ranking table (Table 2) and an inverted triangle table (Table S2). According to the analysis results, CsA combined with LEF ranked first, TwHF combined with LEF ranked second, TwHF combined with MTX ranked third, and TwHF used singly ranked fourth (Figure S5C).

#### 3.2.4. Adverse Events

In the analysis of incidence of adverse events, 13 literature studies were included, involving a total of 3,415 patients, including 11 interventions: MTX, TwHF, TwHF combined with MTX, LEF, TwHF combined with LEF, SSZ combined with MTX, CsA, CsA combined with LEF, FK506 and placebo (Figure S4D). Incidence of adverse events was evaluated by drawing a cumulative probability diagram, a probability efficacy ranking table (Table 2), and an inverted triangle table (Table S3). According to the analysis results, TwHF and TwHF combined with MTX, ranked first and second, respectively (Figure S5D).

#### 3.2.5. Forest Plots

In this study, a forest plot was drawn to assess for inconsistency, as shown in Figure S6A through to S6D. With exception of the M-S-T closed loop with ACR20 as the endpoint, there was no obvious inconsistency in all other closed loops. After analyzing the literature included in the M-S-T closed loop with ACR20 as the endpoint, it is considered that the sources of inconsistency may include different treatment time, different drug doses, heterogeneity caused by allowable adjuvant drugs.

#### 3.2.6. Publication Bias

In addition, this study also evaluated publication bias with funnel plots (Figure S7A through to S7D). The scatters in the 4 funnel plots were almost symmetrical visually, and occasionally a small number of scatters were slightly less symmetrical, indicating that the publication bias in the included studies was overall satisfactory.

## 4. Discussion

TwHF is considered one of the most effective traditional Chinese herbal medicines against rheumatoid arthritis. Extracts of TwHF have been used for hundreds of years in China to treat various symptoms and, over the past 30 years, extracts of TwHF have become a standard therapy for rheumatoid arthritis in China. An earlier meta-analysis on treating RA bone destruction with TwHF was conducted by the team, and the results showed that the TwHF group was superior to the positive drugs MTX and SSZ used in the control group in Van der Heijde modified total sharp score (mTSS), joint erosion (JE), and joint space narrowing (JSN) on X-ray films, with statistical differences (*P* < 0.01). In the aspects of mTSS, joint erosion, and joint space narrowing, TwHF is better than MTX and SSZ. The analysis results showed that TwHF can effectively delay the bone destruction process of RA [5]. Network meta-analysis is a further development and extension of traditional meta-analysis. The biggest advantage of NMA is that it can evaluate different interventions for the treatment of similar diseases for quantitative statistical analysis and comparison. In recent years, the number of NMAs published in various journals and magazines has increased to provide guidance for clinicians in choosing effective interventions. In a previous NMA analysis that was conducted on the efficacy and safety of using DMARDs singly represented by TwHF in the treatment of RA, we found that TwHF is safe and effective [6]. This study provides an updated evaluation based on the results of previous research and with additional interventions, including combined medications. Based on our results on ACR20 response, we found that TwHF combined with MTX had the greatest probability of having the best efficacy among the treatment schemes involved, and TwHF used singly was the second best in the scheme of rankings. The efficacy ranking from best performing to the least are listed as the following: 1^st^ rank TwHF combined with MTX, 2^nd^ rank TwHF, 3^rd^ rank CsA, 4^th^ rank CsA combined with LEF, 5^th^ rank FK506, 6^th^ rank SSZ combined with MTX, 7^th^ rank MINO, 8^th^ rank TwHF combined with LEF, 9^th^ rank MTX, 10^th^ rank LEF, 11^th^ rank SSZ, and 12^th^ rank placebo. Based on our results on ACR50 response, the analysis showed that TwHF combined with MTX ranked second only to CsA combined with LEF, while TwHF ranked third. The detailed ranking list is as follows: 1^st^ rank CsA combined with LEF, 2^nd^ rank TwHF combined with MTX, 3^rd^ rank TwHF, 4^th^ rank TwHF combined with LEF, 5^th^ rank SSZ combined with MTX, 6^th^ rank MTX, 7^th^ rank LEF, 8^th^ rank CsA, 9^th^ rank FK506, 10^th^ rank SSZ, and 11^th^ rank placebo. Based on our results on ACR70 response, the analysis showed that CsA combined with LEF ranked first, TwHF combined with LEF ranked second, TwHF combined with MTX ranked third, and TwHF used singly ranked 4^th^. The detailed rankings are as follows: 1^st^ rank CsA combined with LEF, 2^nd^ rank TwHF combined with LEF, 3^rd^ rank TwHF combined with MTX, 4^th^ rank TwHF, 5^th^ rank CsA, 6^th^ rank LEF, 7^th^ rank SSZ combined with MTX, 8^th^ rank FK506, 9^th^ rank MTX, 10^th^ rank SSZ, and 11^th^ rank placebo. In the analysis of incidence of adverse events, we found the least possibility of incidence with TwHF used singly, followed by TwHF combined with MTX, ranking first and second, respectively. The details of the interventions are as follows: 1^st^ rank TwHF, 2^nd^ rank TwHF combined with MTX, 3^rd^ rank CsA combined with LEF, 4^th^ rank SSZ, 5^th^ rank CsA, 6^th^ rank MTX, 7^th^ rank LEF, 8^th^ rank SSZ combined with MTX, 9^th^ rank TwHF combined with LEF, 10^th^ rank FK506, and 11^th^ rank placebo. In conclusion, from the results of the current analysis it can be considered that compared with the DMARDs currently used to treat RA, TwHF has shown a clear efficacy in treatment of RA, and TwHF combined with MTX performed well under various endpoints. In the ACR20, ACR50, and ACR70 responses, the analysis showed that the efficacy of combination therapy of TwHF was better than its monotherapy. In the ACR20 and ACR50 responses, both monotherapy and combination therapy of TwHF were found to have good efficacy. In analysis of the incidence of adverse events, we found the least possibility of incidence with TwHF used singly. This study also has some limitations. For example, due to the insufficient number of studies, it was not feasible to assess for different dosages of the same drug across different treatment schemes, which may impact the study's results. In addition, some of the included literature studies do not explicitly mention details of randomization method or method of blinding; thus, there is a risk of publication bias. In clinical practice, TwHF is often considered to possess liver and kidney toxicity and to easily cause adverse events. While our study found that TwHF has little possibility of incidence in adverse events, we cannot completely exclude the possibility of publication bias or selective reporting. Thus further review after the publication of more rigorous, high-quality RCTs is warranted.

## 5. Conclusions

This NMA found that in assessment of ACR20 response, TwHF combined with MTX had the greatest probability of achieving the best efficacy among the treatment schemes involved, while TwHF used singly was ranked as second best. In assessment of ACR50 response, the efficacy of TwHF combined with MTX ranked second only to CsA combined with LEF, while TwHF used singly ranked third. In assessment of ACR70 response, CsA combined with LEF ranked first, TwHF combined with LEF ranked second, TwHF combined with MTX ranked third, while TwHF alone ranked fourth. In analysis of incidence of adverse events, the possibility of incidence ranked the lowest with TwHF used singly and the second lowest with TwHF combined with MTX. In conclusion, it can be considered that compared with the DMARDs currently used to treat RA, TwHF has a clear efficacy on RA. Among all treatments, the monotherapy of TwHF and the combination therapy of TwHF and MTX performed well at various endpoints.

In a previous study [6] we conducted an NMA analysis on the efficacy and safety of TwHF and traditional synthetic DMARDs monotherapy in RA. The results indicated that in the direct comparison, TwHF was better than sulphasalazine in ACR 20, ACR 50, and ACR 70 responses; TwHF was superior to placebo in ACR 20 and ACR 50 responses. In indirect comparison, TwHF was superior to MTX, LEF, FK506, MINO, and placebo in ACR 20 response. In the efficacy ranking, TwHF ranked first in ACR 20 and ACR 50 response, and was the preferred treatment. Also, in ACR 70 response, TwHF ranked second (57.8%), second only to LEF (69.6%), which confirmed its efficacy and safety in RA. In clinical practice, combination therapy is also our conventional treatment for RA. Therefore, in this study, based on the previous research, we performed an updated NMA on monotherapy and combination therapy of TwHF and conventional synthetic DMARDs in RA. The research results showed that the clinical protocol of TwHF combination therapy for RA is more in line with clinical practice and has more advantages than other clinical protocols of conventional synthetic DMARD drugs in RA. TwHF can be considered as a potential first-line DMARD for the treatment of RA, but high-quality randomized trial data are still needed to guide the use of TwHF in clinical RA treatment.

## Figures and Tables

**Table 1:** Literature characteristics.

	Intervention			Endpoint	Average age(Years old)	Gender(%F)	Duration oftreatment	Samplesize
	Treatmentgroup	Controlgroup	Othergroup					
Reece, 2002 [16]	L	M		ACR20	L:60 M:61	total:54	16 weeks	39
Cohen, 2001 [17]	L	M		ACR20, 50, 70	L:54 M:53	total:73	48 weeks	380
Lv, 2015 [1]	T	M	*M* + *T*	ACR20, 50, 70	T:51.3 M:51.0 *M* + *T*:50.6	T:81.2 M:85.5 *M* + *T*:79.7	24 weeks	207
Goldbach-mansky R,2009 [18]	S	T		ACR20, 50, 70	T:54 S:52	T:73 S:87	24 weeks	121
Strand，1994 [19]	L	M	P	ACR20, 50, 70	L:54.1 M:53.3 P:54.6	L:72.5 M:75.3 P:70.3	52 weeks	482
Emery，2000 [20]	L	M		ACR20	L:58.3 M:57.8	L:70.7 M:71.3	52 weeks	999
Kraan，2000a [21]	L	M		ACR20, 50	L:60 M:59	L:43.8 M:52.6	16 weeks	35
Kraan，2000b [22]	L	M		ACR20, 50	L:63 M:66	L:57.1 M:37.5	16 weeks	15
Bao, 2003 [23]	L	M		ACR20	L:46.59 M:45.81	L:81.1 M:79.8	12 weeks	504
Capell, 2007 [24]	S	M	*M* + *S*	ACR20, 50, 70	S:55 M:53 *M* + *S*:56	S:75 M:79 *M* + *S*:75	48 weeks	165
Haagsm, 1997 [25]	S	M	*M* + *S*	ACR20	S:56.8 M:54.9 *M* + *S*:57.0	S:61.8 M:65.7 *M* + *S*:66.7	52 weeks	105
Dougads, 1999 [26]	S	M	*M* + *S*	ACR20	S:52 M:50 *M* + *S*:52	S:71 M:74 *M* + *S*:77	52 weeks	205
Smolen, 1999 [27]	L	S	P	ACR 20, 50	S:58.9 L:58.3 P:58.8	S:69 L:76 P:75	24 weeks	358
Karanikolas, 2006 [28]	C	L	L + *C*	ACR20, 50, 70	—	—	48 weeks	102
Scott, 2001 [29]	L	S		ACR20, 50, 70	S:59 L:58	S:69 L:76	24 weeks	262
Yocum,2003 [30]	F	P		ACR20, 50	F:55.9 P:55.8	F:77.2 P:75.8	24 weeks	464
Kawai,2011 [31]	F	P		ACR20, 50, 70	F:47.1 P:50.0	F:90.2 P:80.6	28 weeks	123
Pillemer, 1997 [32]	Mi	P		ACR20	Mi:55.0 P:53.5	Mi:76 P:80	48 weeks	219
Chao-yang Long, 2019 [33]	T	M		ACR20, 50	T:65.03 M:64.79	T:73.3 M:80.0	12 weeks	60
Yong-qiang Wang, 2013 [34]	M	*M* + *T*		ACR20, 50	total:43.4	total:55.6	12 weeks	126
Ming-li Zhao, 2017 [35]	L + *T*	L		ACR20, 50, 70	L:62.24 L + *T*:64.32	L:72.4 L + *T*:83.4	12 weeks	68

TwHF, *Tripterygium wilfordii* Hook F; MTX, methotrexate; LEF, leflunomide; SSZ, sulfasalazine; CsA, cyclosporine; FK506, tacrolimus; and MINO, minocycline; M, MTX; T, TwHF; M + T, TwHF combined with MTX; L, LEF; L + T, TwHF combined with LEF; S, SSZ; M + S, SSZ combined with MTX; C, CsA; L + C, CsA combined with LEF; F, FK506; Mi, MINO; P, placebo.

**Table 2:** Ranking probability of different conventional synthetic DMARDs.

Treatment	ACR20		ACR50		ACR70		Adverse events	
	SUCRA	Rank	SUCRA	Rank	SUCRA	Rank	SUCRA	Rank
*T*	0.749	2	0.726	3	0.606	3	0.107	11
*M* + *T*	0.867	1	0.87	2	0.646	2	0.146	10
*M*	0.371	9	0.457	6	0.261	9	0.441	6
*M* + *S*	0.603	6	0.603	5	0.457	7	0.616	4
*L*	0.263	10	0.397	7	0.508	6	0.524	5
*L* + *T*	0.397	8	0.607	4	0.852	4	0.723	3
L + *C*	0.661	4	0.95	1	0.915	1	0.352	9
*C*	0.664	3	0.374	8	0.542	5	0.415	7
*S*	0.245	11	0.246	10	0.156	10	0.403	8
*F*	0.639	5	0.254	9	0.428	8	0.836	2
Mi	0.505	7	—	—	—	—	—	—
*P*	0.035	12	0.016	11	0.131	11	0.936	1

TwHF, *Tripterygium wilfordii* Hook F; MTX, methotrexate, LEF; leflunomide; SSZ, sulfasalazine; CsA, cyclosporine; FK506, tacrolimus; and MINO, minocycline; M, MTX; T, TwHF; *M* + *T*, TwHF combined with MTX; L, LEF; L + *T*, TwHF combined with LEF; S, SSZ; *M* + *S*, SSZ combined with MTX; C, CsA; L + *C*; CsA combined with LEF; F, FK506; Mi, MINO; P, placebo.

**Table 3:** Inverted triangle table based on ACR20

OR (95%CI)	OR (95%CI)	OR (95%CI)	OR (95%CI)	OR (95%CI)	OR (95%CI)	OR (95%CI)	OR (95%CI)	OR (95%CI)	OR (95%CI)	OR (95%CI)	OR (95%CI)
*M*	1.78 (1.01, 3.14)	2.28 (1.23, 4.21)	0.90 (0.68, 1.17)	1.01 (0.34, 3.02)	0.87 (0.60, 1.26)	1.38 (0.82, 2.35)	1.67 (0.47, 5.91)	1.67 (0.47, 5.91)	1.49 (0.71, 3.13)	1.22 (0.49, 3.01)	0.56 (0.34, 0.92)
**0.56 (0.32, 0.99)**	*T*	1.28 (0.62, 2.63)	0.50 (0.27, 0.92)	0.57 (0.17, 1.93)	0.49 (0.27, 0.89)	0.78 (0.37, 1.63)	0.94 (0.24, 3.72)	0.94 (0.24, 3.72)	0.84 (0.33, 2.09)	0.69 (0.24, 1.96)	0.31 (0.15, 0.65)
**0.44 (0.24, 0.81)**	0.78 (0.38, 1.60)	*M* + *T*	0.39 (0.20, 0.76)	0.44 (0.13, 1.55)	0.38 (0.19, 0.77)	0.61 (0.27, 1.35)	0.73 (0.18, 2.98)	0.73 (0.18, 2.98)	0.65 (0.25, 1.70)	0.53 (0.18, 1.59)	0.24 (0.11, 0.54)
1.12 (0.85, 1.46)	**1.99 (1.08, 3.64)**	**2.55 (1.31, 4.95)**	*L*	1.13 (0.39, 3.26)	0.97 (0.67, 1.40)	1.54 (0.88, 2.70)	1.86 (0.54, 6.41)	1.86 (0.54, 6.41)	1.66 (0.80, 3.45)	1.36 (0.56, 3.32)	0.62 (0.39, 1.00)
0.99 (0.33, 2.98)	1.77 (0.52, 6.01)	2.26 (0.64, 7.94)	0.89 (0.31, 2.58)	*L* + *T*	0.86 (0.28, 2.66)	1.37 (0.41, 4.57)	1.65 (0.32, 8.46)	1.65 (0.32, 8.46)	1.48 (0.41, 5.37)	1.21 (0.30, 4.85)	0.55 (0.17, 1.78)
1.15 (0.79, 1.67)	**2.05 (1.12, 3.74)**	**2.63 (1.30, 5.28)**	1.03 (0.71, 1.49)	1.16 (0.38, 3.58)	*S*	1.59 (0.94, 2.69)	1.92 (0.53, 6.98)	1.92 (0.53, 6.98)	1.71 (0.80, 3.69)	1.40 (0.56, 3.53)	0.64 (0.38, 1.09)
0.72 (0.43, 1.23)	1.29 (0.61, 2.70)	1.65 (0.74, 3.67)	0.65 (0.37, 1.13)	0.73 (0.22, 2.43)	0.63 (0.37, 1.06)	*M* + *S*	1.20 (0.31, 4.69)	1.20 (0.31, 4.69)	1.08 (0.45, 2.60)	0.88 (0.32, 2.45)	0.40 (0.20, 0.80)
0.60 (0.17, 2.13)	1.07 (0.27, 4.24)	1.37 (0.34, 5.58)	0.54 (0.16, 1.86)	0.61 (0.12, 3.10)	0.52 (0.14, 1.90)	0.83 (0.21, 3.23)	*C*	1.00 (0.26, 3.79)	0.89 (0.21, 3.76)	0.73 (0.16, 3.37)	0.33 (0.09, 1.26)
0.60 (0.17, 2.13)	1.07 (0.27, 4.24)	1.37 (0.34, 5.58)	0.54 (0.16, 1.86)	0.61 (0.12, 3.10)	0.52 (0.14, 1.90)	0.83 (0.21, 3.23)	1.00 (0.26, 3.79)	*L* + *C*	0.89 (0.21, 3.76)	0.73 (0.16, 3.37)	0.33 (0.09, 1.26)
0.67 (0.32, 1.42)	1.20 (0.48, 2.99)	1.53 (0.59, 4.00)	0.60 (0.29, 1.25)	0.68 (0.19, 2.46)	0.58 (0.27, 1.26)	0.93 (0.38, 2.25)	1.12 (0.27, 4.71)	1.12 (0.27, 4.71)	*F*	0.82 (0.32, 2.09)	0.37 (0.22, 0.65)
0.82 (0.33, 2.02)	1.46 (0.51, 4.17)	1.87 (0.63, 5.55)	0.73 (0.30, 1.79)	0.83 (0.21, 3.32)	0.71 (0.28, 1.79)	1.13 (0.41, 3.15)	1.37 (0.30, 6.28)	1.37 (0.30, 6.28)	1.22 (0.48, 3.11)	Mi	0.46 (0.22, 0.97)
**1.79 (1.09, 2.95)**	**3.19 (1.54, 6.63)**	**4.09 (1.87, 8.96)**	1.61 (1.00, 2.59)	1.81 (0.56, 5.81)	1.56 (0.92, 2.65)	**2.48 (1.25, 4.94)**	2.99 (0.79, 11.26)	2.99 (0.79, 11.26)	**2.67 (1.54, 4.64)**	**2.19 (1.03, 4.65)**	*P*

Weighted mean difference with 95% CIs of network meta-analysis. Treatments are reported in alphabetical order. Results of direct comparisons are listed in the lower-left triangle, and the estimation is calculated as the row-defining treatment compared with the column-defining treatment. Results of network meta-analysis are listed in the upper-right triangle, and the estimation is calculated as the column-defining treatment compared with the row-defining treatment. Bolding indicates that the difference has a statistical significance.

## Supplementary Materials

Supplementary Materials Figure S1: PRISMA-2009-Flow-Diagram-MS-Word: PRISMA flowchart. Figure S2: Risk of bias graph. Figure S3: Risk of bias summary. Figure S4: The cumulative probability diagram. A. With ACR20 as the endpoint. B. With ACR50 as the endpoint. C. With ACR70 as the endpoint. D. The analysis of adverse events. Figure S5: Forest plots. A. With ACR20 as the endpoint. B. With ACR50 as the endpoint. C. With ACR70 as the endpoint. D. The analysis of adverse events. Figure S6: Inconsistent assessment. A. With ACR20 as the endpoint. B. With ACR50 as the endpoint. C. With ACR70 as the endpoint. D. The analysis of adverse events. Figure S7: The publication bias. A. With ACR20 as the endpoint. B. With ACR50 as the endpoint. C. With ACR70 as the endpoint. D. The analysis of adverse events. Table S1: Inverted triangle table based on ACR50. Table S2: Inverted triangle table based on ACR70. Table S3: Inverted triangle table based on adverse events. Table S4: Search strategy.
