# Supplementary material for: A Systematic Review and Network Meta-Analysis about the Efficacy and Safety of Tripterygium wilfordii Hook F in Rheumatoid Arthritis
Source: Evid Based Complement Alternat Med. 2022 May 10;2022:3181427. doi: 10.1155/2022/3181427 (PMC9113883; doi:10.1155/2022/3181427)
Supplement: Supplementary Materials — Figure S1: PRISMA-2009-Flow-Diagram-MS-Word: PRISMA flowchart. Figure S2: Risk of bias graph. Figure S3: Risk of bias summary. Figure S4: The cumulative probability diagram. A. With ACR20 as the endpoint. B. With ACR50 as the endpoint. C. With ACR70 as the endpoint. D. The analysis of adverse events. Figure S5: Forest plots. A. With ACR20 as the endpoint. B. With ACR50 as the endpoint. C. With ACR70 as the endpoint. D. The analysis of adverse events. Figure S6: Inconsistent assessment. A. With ACR20 as the endpoint. B. With ACR50 as the endpoint. C. With ACR70 as the endpoint. D. The analysis of adverse events. Figure S7: The publication bias. A. With ACR20 as the endpoint. B. With ACR50 as the endpoint. C. With ACR70 as the endpoint. D. The analysis of adverse events. Table S1: Inverted triangle table based on ACR50. Table S2: Inverted triangle table based on ACR70. Table S3: Inverted triangle table based on adverse events. Table S4: Search strategy. [file 3181427.f1.zip › 3181427.f1/Figure S3.pdf]

	Random sequence generation (selection bias)	Allocation concealment (selection bias)	Blinding of participants and personnel (performance bias)	Blinding of outcome assessment (detection bias)	Incomplete outcome data (attrition bias)	Selective reporting (reporting bias)	Other bias
Bao 2003	+	+	+	+	?	?	?
Capell 2007	+	?	+	+	?	?	?
Cohen 2001	+	+	+	?	?	?	?
Dougados 1999	+	+	+	+	?	?	?
Emery 2000	+	+	?	+	?	?	?
Gondbach-Mansky R 2009	+	?	−	−	?	?	?
Haagsma 1997	+	?	+	?	?	?	?
Karanikolas 2006	+	?	?	?	+	+	?
Kawai 2011	+	?	?	?	?	?	?
Kraan MC 2000a	+	?	+	?	?	?	?
Kraan MC 2000b	+	?	+	?	?	?	?
Long 2019	+	?	?	−	?	?	?
Lv 2014	+	?	−	+	+	+	?
Pillemer 1997	+	?	?	?	?	?	?
Reece 2002	+	+	+	+	?	?	?
Scott 2001	+	+	+	+	?	?	?
Smolen JS 1999	+	?	+	?	?	?	?
Strand V 1999	+	+	+	+	+	+	+
Wang 2013	+	?	?	?	?	?	?
Yocum 2003	+	?	?	?	?	?	?
Zhao 2017	+	?	?	?	?	?	?
