# Supplementary material for: A Systematic Review and Network Meta-Analysis about the Efficacy and Safety of Tripterygium wilfordii Hook F in Rheumatoid Arthritis
Source: Evid Based Complement Alternat Med. 2022 May 10;2022:3181427. doi: 10.1155/2022/3181427 (PMC9113883; doi:10.1155/2022/3181427)
Supplement: Supplementary Materials — Figure S1: PRISMA-2009-Flow-Diagram-MS-Word: PRISMA flowchart. Figure S2: Risk of bias graph. Figure S3: Risk of bias summary. Figure S4: The cumulative probability diagram. A. With ACR20 as the endpoint. B. With ACR50 as the endpoint. C. With ACR70 as the endpoint. D. The analysis of adverse events. Figure S5: Forest plots. A. With ACR20 as the endpoint. B. With ACR50 as the endpoint. C. With ACR70 as the endpoint. D. The analysis of adverse events. Figure S6: Inconsistent assessment. A. With ACR20 as the endpoint. B. With ACR50 as the endpoint. C. With ACR70 as the endpoint. D. The analysis of adverse events. Figure S7: The publication bias. A. With ACR20 as the endpoint. B. With ACR50 as the endpoint. C. With ACR70 as the endpoint. D. The analysis of adverse events. Table S1: Inverted triangle table based on ACR50. Table S2: Inverted triangle table based on ACR70. Table S3: Inverted triangle table based on adverse events. Table S4: Search strategy. [file 3181427.f1.zip › 3181427.f1/Table S1.Inverted triangle table based on ACR50.docx]

Table S1: Inverted triangle table based on ACR50

| **OR (95%CI)** | **OR (95%CI)** | | **OR (95%CI)** | | **OR (95%CI)** | | **OR (95%CI)** | | **OR (95%CI)** | | **OR (95%CI)** | | **OR (95%CI)** | | **OR (95%CI)** | | **OR (95%CI)** | | **OR (95%CI)** |
| --- | --- | --- | --- | --- | --- | --- | --- | --- | --- | --- | --- | --- | --- | --- | --- | --- | --- | --- | --- |
| M | | 1.88 (0.96,3.70) | | 2.92 (1.53,5.55) | | 0.90 (0.55,1.48) | | 1.45 (0.42,5.03) | | 0.69 (0.35,1.34) | | 1.47 (0.40,5.34) | | 0.84 (0.25,2.87) | | 5.04 (1.35,18.83) | 0.64 (0.23,1.77) | 0.32 (0.15,0.67) | |
| 0.53 (0.27,1.04) | | T | | 1.55 (0.71,3.36) | | 0.48 (0.21,1.07) | | 0.77 (0.19,3.11) | | 0.36 (0.15,0.91) | | 0.78 (0.18,3.31) | | 0.45 (0.11,1.78) | | 2.67 (0.62,11.56) | 0.34 (0.10,1.13) | 0.17 (0.06,0.45) | |
| **0.34 (0.18,0.65)** | | 0.65 (0.30,1.40) | | M+T | | 0.31 (0.14,0.69) | | 0.50 (0.12,2.01) | | 0.23 (0.09,0.59) | | 0.50 (0.12,2.12) | | 0.29 (0.07,1.14) | | 1.73 (0.40,7.44) | 0.22 (0.07,0.73) | 0.11 (0.04,0.29) | |
| 1.11 (0.68,1.83) | | 2.09 (0.93,4.70) | | **3.25 (1.45,7.25)** | | L | | 1.62 (0.52,5.04) | | 0.76 (0.44,1.32) | | 1.63 (0.44,6.10) | | 0.93 (0.30,2.87) | | 5.60 (1.65,18.99) | 0.71 (0.27,1.86) | 0.36 (0.19,0.68) | |
| 0.69 (0.20,2.38) | | 1.30 (0.32,5.24) | | 2.01 (0.50,8.10) | | 0.62 (0.20,1.93) | | L+T | | 0.47 (0.13,1.67) | | 1.01 (0.18,5.77) | | 0.58 (0.12,2.86) | | 3.47 (0.65,18.41) | 0.44 (0.10,1.95) | 0.22 (0.06,0.82) | |
| 1.46 (0.75,2.84) | | **2.75 (1.10,6.85)** | | **4.26 (1.70,10.63)** | | 1.31 (0.76,2.27) | | 2.12 (0.60,7.50) | | S | | 2.14 (0.58,7.94) | | 1.22 (0.35,4.27) | | 7.34 (1.93,28.01) | 0.93 (0.33,2.60) | 0.47 (0.22,0.97) | |
| 0.68 (0.19,2.48) | | 1.28 (0.30,5.46) | | 1.99 (0.47,8.39) | | 0.61 (0.16,2.29) | | 0.99 (0.17,5.66) | | 0.47 (0.13,1.73) | | M+S | | 0.57 (0.10,3.24) | | 3.43 (0.57,20.73) | 0.43 (0.09,2.13) | 0.22 (0.05,0.90) | |
| 1.19 (0.35,4.07) | | 2.24 (0.56,8.95) | | 3.48 (0.87,13.84) | | 1.07 (0.35,3.30) | | 1.73 (0.35,8.57) | | 0.82 (0.23,2.85) | | 1.75 (0.31,9.89) | | C | | 6.00 (1.76,20.50) | 0.76 (0.17,3.34) | 0.38 (0.10,1.40) | |
| **0.20 (0.05,0.74)** | | 0.37 (0.09,1.62) | | 0.58 (0.13,2.50) | | **0.18 (0.05,0.61)** | | 0.29 (0.05,1.53) | | **0.14 (0.04,0.52)** | | 0.29 (0.05,1.76) | | **0.17 (0.05,0.57)** | | L+C | 0.13 (0.03,0.60) | 0.06 (0.02,0.25) | |
| 1.57 (0.56,4.38) | | 2.96 (0.89,9.87) | | **4.59 (1.38,15.29)** | | 1.41 (0.54,3.72) | | 2.28 (0.51,10.18) | | 1.08 (0.39,3.02) | | 2.31 (0.47,11.30) | | 1.32 (0.30,5.81) | | **7.92 (1.67,37.61)** | F | 0.50 (0.25,1.03) | |
| **3.12 (1.50,6.51)** | | **5.89 (2.23,15.51)** | | **9.12 (3.47,23.99)** | | **2.81 (1.47,5.38)** | | **4.54 (1.22,16.84)** | | **2.14 (1.03,4.48)** | | **4.58 (1.11,18.93)** | | 2.62 (0.72,9.60) | | **15.74 (3.95,62.75)** | 1.99 (0.97,4.07) | P | |

Weighted mean difference with 95% CIs of network meta-analysis. Treatments are reported in alphabetical order. Results of direct comparisons are listed in the lower-left triangle, and the estimation is calculated as the row-defining treatment compared with the column-defining treatment. Results of network meta-analysis are listed in the upper-right triangle, and the estimation is calculated as the column-defining treatment compared with the row-defining treatment. Bold indicates that the difference has a statistical significance.

TwHF: *Tripterygium wilfordii* Hook F, MTX: methotrexate, LEF: leflunomide, SSZ: sulphasalazine, CsA: cyclosporine, FK506: tacrolimus, and MINO: minocycline. *M:MTX T:TwHF M+T:TwHF combined with MTX L:LEF L+T:TwHF combined with LEF S:SSZ M+S:SSZ combined with MTX C:CsA L+C:CsA combined with LEF F:FK5O6 Mi:MINO P:placebo.*
