# Supplementary material for: A Systematic Review and Network Meta-Analysis about the Efficacy and Safety of Tripterygium wilfordii Hook F in Rheumatoid Arthritis
Source: Evid Based Complement Alternat Med. 2022 May 10;2022:3181427. doi: 10.1155/2022/3181427 (PMC9113883; doi:10.1155/2022/3181427)
Supplement: Supplementary Materials — Figure S1: PRISMA-2009-Flow-Diagram-MS-Word: PRISMA flowchart. Figure S2: Risk of bias graph. Figure S3: Risk of bias summary. Figure S4: The cumulative probability diagram. A. With ACR20 as the endpoint. B. With ACR50 as the endpoint. C. With ACR70 as the endpoint. D. The analysis of adverse events. Figure S5: Forest plots. A. With ACR20 as the endpoint. B. With ACR50 as the endpoint. C. With ACR70 as the endpoint. D. The analysis of adverse events. Figure S6: Inconsistent assessment. A. With ACR20 as the endpoint. B. With ACR50 as the endpoint. C. With ACR70 as the endpoint. D. The analysis of adverse events. Figure S7: The publication bias. A. With ACR20 as the endpoint. B. With ACR50 as the endpoint. C. With ACR70 as the endpoint. D. The analysis of adverse events. Table S1: Inverted triangle table based on ACR50. Table S2: Inverted triangle table based on ACR70. Table S3: Inverted triangle table based on adverse events. Table S4: Search strategy. [file 3181427.f1.zip › 3181427.f1/Table S2. Inverted triangle table based on ACR70.docx]

Table S2: Inverted triangle table based on ACR70

| **OR (95%CI)** | **OR (95%CI)** | **OR (95%CI)** | **OR (95%CI)** | **OR (95%CI)** | **OR (95%CI)** | **OR (95%CI)** | **OR (95%CI)** | **OR (95%CI)** | **OR (95%CI)** | **OR (95%CI)** |
| --- | --- | --- | --- | --- | --- | --- | --- | --- | --- | --- |
| M | 2.49 (0.74,8.42) | 2.86 (0.80,10.27) | 1.87 (0.77,4.55) | 6.90 (1.16,40.94) | 0.66 (0.19,2.37) | 1.61 (0.16,15.84) | 2.22 (0.40,12.28) | 9.29 (1.67,51.69) | 1.41 (0.20,10.25) | 0.59 (0.13,2.66) |
| 0.40 (0.12,1.35) | T | 1.15 (0.32,4.06) | 0.75 (0.18,3.12) | 2.77 (0.34,22.61) | 0.27 (0.06,1.12) | 0.65 (0.05,7.77) | 0.89 (0.12,6.86) | 3.72 (0.48,28.83) | 0.57 (0.06,5.31) | 0.24 (0.04,1.57) |
| 0.35 (0.10,1.26) | 0.87 (0.25,3.09) | M+T | 0.65 (0.14,2.98) | 2.41 (0.28,20.98) | 0.23 (0.04,1.21) | 0.56 (0.04,7.34) | 0.78 (0.09,6.37) | 3.25 (0.39,26.80) | 0.49 (0.05,5.01) | 0.21 (0.03,1.45) |
| 0.53 (0.22,1.30) | 1.33 (0.32,5.54) | 1.53 (0.34,6.95) | L | 3.68 (0.79,17.25) | 0.35 (0.11,1.20) | 0.86 (0.08,9.06) | 1.19 (0.27,5.12) | 4.96 (1.14,21.56) | 0.76 (0.11,5.43) | 0.32 (0.07,1.37) |
| **0.14 (0.02,0.86)** | 0.36 (0.04,2.95) | 0.41 (0.05,3.61) | 0.27 (0.06,1.27) | L+T | 0.10 (0.01,0.69) | 0.23 (0.01,3.90) | 0.32 (0.04,2.70) | 1.35 (0.16,11.34) | 0.21 (0.02,2.51) | 0.09 (0.01,0.72) |
| 1.51 (0.42,5.39) | 3.76 (0.89,15.84) | 4.31 (0.83,22.43) | 2.82 (0.84,9.51) | **10.39 (1.46,74.13)** | S | 2.43 (0.25,23.86) | 3.34 (0.50,22.39) | 13.99 (2.08,94.18) | 2.13 (0.22,20.24) | 0.89 (0.14,5.56) |
| 0.62 (0.06,6.08) | 1.54 (0.13,18.55) | 1.77 (0.14,23.04) | 1.16 (0.11,12.20) | 4.27 (0.26,71.27) | 0.41 (0.04,4.04) | M+S | 1.38 (0.09,21.95) | 5.75 (0.36,92.17) | 0.88 (0.04,17.30) | 0.37 (0.02,5.36) |
| 0.45 (0.08,2.49) | 1.12 (0.15,8.65) | 1.29 (0.16,10.58) | 0.84 (0.20,3.64) | 3.11 (0.37,26.05) | 0.30 (0.04,2.00) | 0.73 (0.05,11.60) | C | 4.18 (0.97,18.08) | 0.64 (0.05,7.42) | 0.27 (0.03,2.12) |
| **0.11 (0.02,0.60)** | 0.27 (0.03,2.08) | 0.31 (0.04,2.54) | **0.20 (0.05,0.88)** | 0.74 (0.09,6.26) | 0.07 (0.01,0.48) | 0.17 (0.01,2.79) | 0.24 (0.06,1.03) | L+C | 0.15 (0.01,1.78) | 0.06 (0.01,0.51) |
| 0.71 (0.10,5.12) | 1.76 (0.19,16.51) | 2.02 (0.20,20.49) | 1.32 (0.18,9.51) | 4.88 (0.40,59.67) | 0.47 (0.05,4.46) | 1.14 (0.06,22.54) | 1.57 (0.13,18.28) | 6.56 (0.56,76.78) | F | 0.42 (0.11,1.52) |
| 1.69 (0.38,7.62) | 4.23 (0.64,28.06) | 4.85 (0.69,34.16) | 3.17 (0.73,13.83) | **11.69 (1.39,98.68)** | 1.13 (0.18,7.04) | 2.74 (0.19,40.15) | 3.76 (0.47,29.96) | 15.74 (1.97,125.96) | 2.40 (0.66,8.74) | P |

Weighted mean difference with 95% CIs of network meta-analysis. Treatments are reported in alphabetical order. Results of direct comparisons are listed in the lower-left triangle, and the estimation is calculated as the row-defining treatment compared with the column-defining treatment. Results of network meta-analysis are listed in the upper-right triangle, and the estimation is calculated as the column-defining treatment compared with the row-defining treatment. Bold indicates that the difference has a statistical significance.

TwHF: *Tripterygium wilfordii* Hook F, MTX: methotrexate, LEF: leflunomide, SSZ: sulphasalazine, CsA: cyclosporine, FK506: tacrolimus, and MINO: minocycline. *M:MTX T:TwHF M+T:TwHF combined with MTX L:LEF L+T:TwHF combined with LEF S:SSZ M+S:SSZ combined with MTX C:CsA L+C:CsA combined with LEF F:FK5O6 Mi:MINO P:placebo*
