# Supplementary material for: A Systematic Review and Network Meta-Analysis about the Efficacy and Safety of Tripterygium wilfordii Hook F in Rheumatoid Arthritis
Source: Evid Based Complement Alternat Med. 2022 May 10;2022:3181427. doi: 10.1155/2022/3181427 (PMC9113883; doi:10.1155/2022/3181427)
Supplement: Supplementary Materials — Figure S1: PRISMA-2009-Flow-Diagram-MS-Word: PRISMA flowchart. Figure S2: Risk of bias graph. Figure S3: Risk of bias summary. Figure S4: The cumulative probability diagram. A. With ACR20 as the endpoint. B. With ACR50 as the endpoint. C. With ACR70 as the endpoint. D. The analysis of adverse events. Figure S5: Forest plots. A. With ACR20 as the endpoint. B. With ACR50 as the endpoint. C. With ACR70 as the endpoint. D. The analysis of adverse events. Figure S6: Inconsistent assessment. A. With ACR20 as the endpoint. B. With ACR50 as the endpoint. C. With ACR70 as the endpoint. D. The analysis of adverse events. Figure S7: The publication bias. A. With ACR20 as the endpoint. B. With ACR50 as the endpoint. C. With ACR70 as the endpoint. D. The analysis of adverse events. Table S1: Inverted triangle table based on ACR50. Table S2: Inverted triangle table based on ACR70. Table S3: Inverted triangle table based on adverse events. Table S4: Search strategy. [file 3181427.f1.zip › 3181427.f1/Table S3.Inverted triangle table based on Safety.docx]

**Table S3:** Inverted triangle table based on Safety

| **OR (95%CI)** | **OR (95%CI)** | **OR (95%CI)** | **OR (95%CI)** | **OR (95%CI)** | **OR (95%CI)** | **OR (95%CI)** | **OR (95%CI)** | **OR (95%CI)** | **OR (95%CI)** | **OR (95%CI)** |
| --- | --- | --- | --- | --- | --- | --- | --- | --- | --- | --- |
| M | 0.44 (0.20,0.97) | 0.49 (0.20,1.16) | 1.16 (0.66,2.03) | 2.42 (0.47,12.44) | 0.94 (0.44,2.00) | 1.66 (0.37,7.44) | 0.95 (0.19,4.59) | 0.81 (0.17,3.87) | 4.68 (0.60,36.32) | 6.75 (1.43,31.99) |
| **2.25 (1.03,4.94)** | T | 1.10 (0.42,2.90) | 2.62 (1.06,6.46) | 5.46 (0.92,32.47) | 2.12 (0.82,5.44) | 3.75 (0.72,19.61) | 2.13 (0.38,12.04) | 1.82 (0.33,10.17) | 10.55 (1.23,90.20) | 15.22 (2.83,81.77) |
| 2.05 (0.87,4.88) | 0.91 (0.35,2.41) | M+T | 2.38 (0.86,6.57) | 4.98 (0.79,31.37) | 1.93 (0.64,5.83) | 3.42 (0.61,19.04) | 1.94 (0.32,11.66) | 1.66 (0.28,9.84) | 9.62 (1.06,87.55) | 13.87 (2.38,80.70) |
| 0.86 (0.49,1.51) | **0.38 (0.15,0.94)** | 0.42 (0.15,1.16) | L | 2.09 (0.45,9.70) | 0.81 (0.41,1.58) | 1.43 (0.31,6.71) | 0.81 (0.19,3.57) | 0.70 (0.16,3.01) | 4.03 (0.55,29.66) | 5.82 (1.32,25.67) |
| 0.41 (0.08,2.12) | 0.18 (0.03,1.09) | 0.20 (0.03,1.27) | 0.48 (0.10,2.23) | L+T | 0.39 (0.07,2.07) | 0.69 (0.08,6.06) | 0.39 (0.05,3.29) | 0.33 (0.04,2.78) | 1.93 (0.16,23.97) | 2.79 (0.33,23.60) |
| 1.07 (0.50,2.27) | 0.47 (0.18,1.22) | 0.52 (0.17,1.57) | 1.24 (0.63,2.41) | 2.58 (0.48,13.78) | S | 1.77 (0.38,8.22) | 1.01 (0.20,5.10) | 0.86 (0.17,4.30) | 4.99 (0.68,36.50) | 7.19 (1.64,31.54) |
| 0.60 (0.13,2.69) | 0.27 (0.05,1.39) | 0.29 (0.05,1.63) | 0.70 (0.15,3.26) | 1.46 (0.16,12.84) | 0.56 (0.12,2.61) | M+S | 0.57 (0.07,4.81) | 0.49 (0.06,4.07) | 2.81 (0.24,33.33) | 4.06 (0.51,32.54) |
| 1.06 (0.22,5.14) | 0.47 (0.08,2.65) | 0.51 (0.09,3.09) | 1.23 (0.28,5.38) | 2.56 (0.30,21.60) | 0.99 (0.20,5.02) | 1.76 (0.21,14.91) | C | 0.86 (0.20,3.62) | 4.95 (0.41,59.29) | 7.14 (0.88,58.00) |
| 1.24 (0.26,5.91) | 0.55 (0.10,3.06) | 0.60 (0.10,3.56) | 1.43 (0.33,6.18) | 2.99 (0.36,24.95) | 1.16 (0.23,5.78) | 2.06 (0.25,17.23) | 1.17 (0.28,4.94) | L+C | 5.79 (0.49,68.60) | 8.35 (1.04,66.98) |
| 0.21 (0.03,1.66) | **0.09 (0.01,0.81)** | **0.10 (0.01,0.95)** | 0.25 (0.03,1.82) | 0.52 (0.04,6.42) | 0.20 (0.03,1.47) | 0.36 (0.03,4.21) | 0.20 (0.02,2.42) | 0.17 (0.01,2.05) | F | 1.44 (0.38,5.47) |
| **0.15 (0.03,0.70)** | **0.07 (0.01,0.35)** | **0.07 (0.01,0.42)** | **0.17 (0.04,0.76)** | 0.36 (0.04,3.04) | **0.14 (0.03,0.61)** | 0.25 (0.03,1.98) | 0.14 (0.02,1.14) | **0.12 (0.01,0.96)** | 0.69 (0.18,2.63) | P |

Weighted mean difference with 95% CIs of network meta-analysis. Treatments are reported in alphabetical order. Results of direct comparisons are listed in the lower-left triangle, and the estimation is calculated as the row-defining treatment compared with the column-defining treatment. Results of network meta-analysis are listed in the upper-right triangle, and the estimation is calculated as the column-defining treatment compared with the row-defining treatment. Bold indicates that the difference has a statistical significance.

TwHF: *Tripterygium wilfordii* Hook F, MTX: methotrexate, LEF: leflunomide, SSZ: sulphasalazine, CsA: cyclosporine, FK506: tacrolimus, and MINO: minocycline. *M:MTX T:TwHF M+T:TwHF combined with MTX L:LEF L+T:TwHF combined with LEF S:SSZ M+S:SSZ combined with MTX C:CsA L+C:CsA combined with LEF F:FK5O6 Mi:MINO P:placebo*
