# Supplementary material for: A Systematic Review and Network Meta-Analysis about the Efficacy and Safety of Tripterygium wilfordii Hook F in Rheumatoid Arthritis
Source: Evid Based Complement Alternat Med. 2022 May 10;2022:3181427. doi: 10.1155/2022/3181427 (PMC9113883; doi:10.1155/2022/3181427)
Supplement: Supplementary Materials — Figure S1: PRISMA-2009-Flow-Diagram-MS-Word: PRISMA flowchart. Figure S2: Risk of bias graph. Figure S3: Risk of bias summary. Figure S4: The cumulative probability diagram. A. With ACR20 as the endpoint. B. With ACR50 as the endpoint. C. With ACR70 as the endpoint. D. The analysis of adverse events. Figure S5: Forest plots. A. With ACR20 as the endpoint. B. With ACR50 as the endpoint. C. With ACR70 as the endpoint. D. The analysis of adverse events. Figure S6: Inconsistent assessment. A. With ACR20 as the endpoint. B. With ACR50 as the endpoint. C. With ACR70 as the endpoint. D. The analysis of adverse events. Figure S7: The publication bias. A. With ACR20 as the endpoint. B. With ACR50 as the endpoint. C. With ACR70 as the endpoint. D. The analysis of adverse events. Table S1: Inverted triangle table based on ACR50. Table S2: Inverted triangle table based on ACR70. Table S3: Inverted triangle table based on adverse events. Table S4: Search strategy. [file 3181427.f1.zip › 3181427.f1/Table S4.search strategy.docx]

Table S4 :PubMed search strategy

| Databases: | PubMed |  |  |  |  |  |  |  | |
| --- | --- | --- | --- | --- | --- | --- | --- | --- | --- |
| Search number | Search query | | | | | | | | Search fields |
| #1 | ((((((((((( Rheumatoid Arthritis )) OR ( Rheumatoid arthrosis ) ) OR ( arthrosis deformans)) OR ( Arthritis deformans )) OR ( beauvais disease)) OR ( Rheumatic Arthritis ) ) OR ( Rheumatic arthrosis ) ) OR ( Rheumatoid polyarthritis ) ) OR (rheumarthritis)) OR (caplan's syndrome)) OR (felty's syndrome)) (272503) | | | | | | | | All fields |
| #2 | ((((((((((((Randomized controlled trial) OR Randomized controlled trials) OR random allocation) OR random allocate) OR randomly allocate) OR double-blind method) OR single-blind method) OR double blind) OR single blind) OR triple blind) OR clinical trial) OR clinical trials) NOT animal (1477276) | | | | | | | | All fields |
| #3 | ((((((((((((((((((((((((tripterygium)) OR (tripterygium hypoglaucum)) OR (tripterygiumhypoglaucums ) ) OR ( tripterygium wilfordii ) ) OR ( tripterygium wilfordius )) OR wilfordius ) ) OR ( Leigong Teng )) OR ( Lei gong Tengs )) OR ( lei gong teng ) ) OR ( leigongteng ) ) OR (Thundergod Vine)) OR (Thundergod Vines)) OR ( thunder god vine)) OR ( thundergodvine)) OR ( Tripterygium)) OR ( Triptolid)) OR (triptolide)) OR (tripterin)) OR (Tripdiolide)) OR (Tripdiolid)) OR (Triptonide)) OR (wilfordine)) OR (tripterin))) (3470) | | | | | | | | All fields |
| #4 | ((((((((((((((Methotrexate) OR mexate) OR Abitrexate) OR Antifolan) OR Enthexate) OR Farmitrexate) OR Folex) OR Ledertrexate) OR Methoblastin) OR Methohexate) OR Methotrate) OR Methylaminopterin) OR Metotrexate Novatrex) OR Rheumatrex) OR MTX (59193) | | | | | | | | All fields |
| #5 | leflunomide OR arava OR isoxazole OR LEF (21803) | | | | | | | | All fields |
| #6 | Sulfasalazine OR Salicylazosulfapyridine OR Sulphasalazine OR Salazosulfapyridine OR Azulfadine OR Salazopyrin OR Pleon OR Azulfidine OR Pyralin EN OR Asulfidine OR Azulfidine EN OR Sulfasalazin medac OR SSZ OR SASP (7658) | | | | | | | | All fields |
| #7 | cyclosporine OR Ciclosporin OR cyclosporine OR cyclosporin OR ciclosporin A OR cyclosporine A OR cyclosporin A OR CsA (70197) | | | | | | | | All fields |
| #8 | tacrolimus OR fujimycin OR Prograf OR Advagraf OR Protopic OR FK506 (26306) | | | | | | | | All fields |
| #9 | Minocycline OR Klinomycin OR Mincmycin OR Minocyn OR Minomax OR Minomyc (8922) | | | | | | | | All fields |
| #10 | Tripterygium wilfordii Hook F OR sulphasalazine OR leflunomide OR Methotrexate OR cyclosporine OR tacrolimus OR Minocycline (140142) | | | | | | | | MeSH Terms |
| #11 | #3 OR #4 OR #5 OR #6 OR #7 OR #8 OR #9 OR #10 (190003) | | | | | | | | All fields |
| #12 | #1 AND #11 (14555) | | | | | | | | All fields |
| #13 | #2 AND #12 (3644) | | | | | | | | All fields |
|  |  | | | | | | | |  |
