# Supplementary figures and images for: A Systematic Review and Network Meta-Analysis about the Efficacy and Safety of Tripterygium wilfordii Hook F in Rheumatoid Arthritis
Source: Evid Based Complement Alternat Med. 2022 May 10;2022:3181427. doi: 10.1155/2022/3181427 (PMC9113883; doi:10.1155/2022/3181427)

Cumulative Probabilities

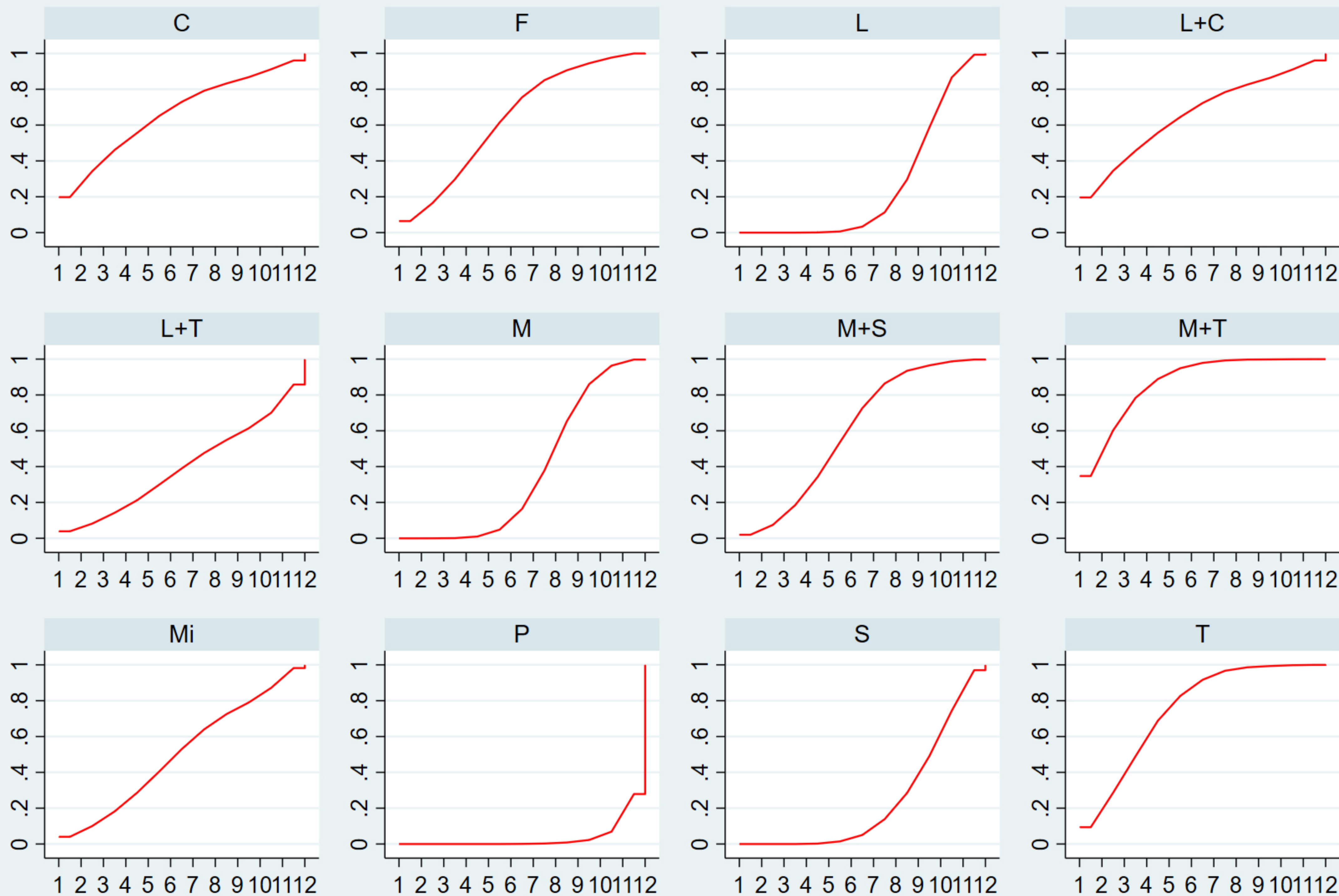

Rank

Graphs by Treatment

Supplement: Supplementary Materials — Figure S1: PRISMA-2009-Flow-Diagram-MS-Word: PRISMA flowchart. Figure S2: Risk of bias graph. Figure S3: Risk of bias summary. Figure S4: The cumulative probability diagram. A. With ACR20 as the endpoint. B. With ACR50 as the endpoint. C. With ACR70 as the endpoint. D. The analysis of adverse events. Figure S5: Forest plots. A. With ACR20 as the endpoint. B. With ACR50 as the endpoint. C. With ACR70 as the endpoint. D. The analysis of adverse events. Figure S6: Inconsistent assessment. A. With ACR20 as the endpoint. B. With ACR50 as the endpoint. C. With ACR70 as the endpoint. D. The analysis of adverse events. Figure S7: The publication bias. A. With ACR20 as the endpoint. B. With ACR50 as the endpoint. C. With ACR70 as the endpoint. D. The analysis of adverse events. Table S1: Inverted triangle table based on ACR50. Table S2: Inverted triangle table based on ACR70. Table S3: Inverted triangle table based on adverse events. Table S4: Search strategy. [file 3181427.f1.zip › 3181427.f1/Figure S4A.pdf]

Cumulative Probabilities

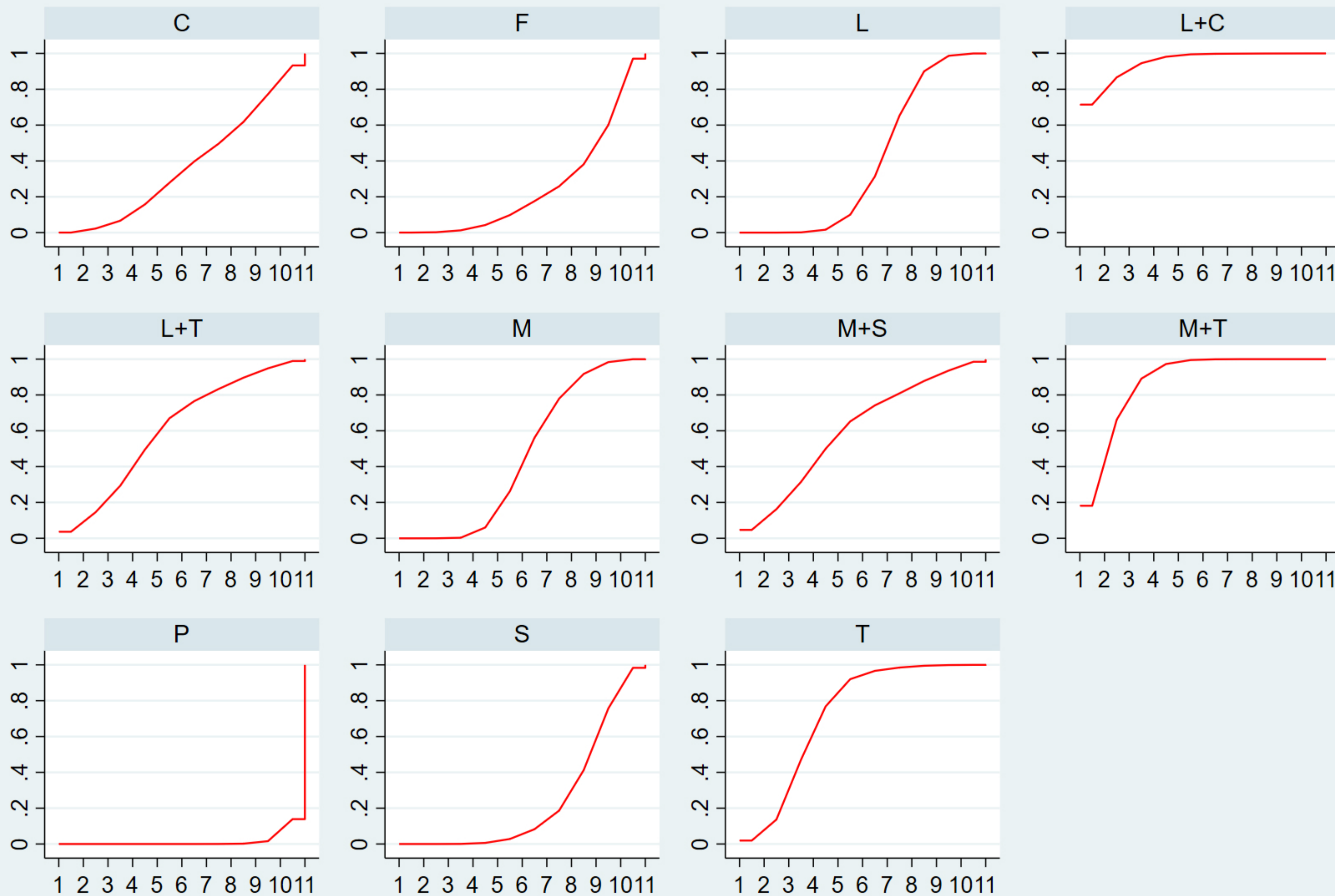

Rank

Graphs by Treatment

Supplement: Supplementary Materials — Figure S1: PRISMA-2009-Flow-Diagram-MS-Word: PRISMA flowchart. Figure S2: Risk of bias graph. Figure S3: Risk of bias summary. Figure S4: The cumulative probability diagram. A. With ACR20 as the endpoint. B. With ACR50 as the endpoint. C. With ACR70 as the endpoint. D. The analysis of adverse events. Figure S5: Forest plots. A. With ACR20 as the endpoint. B. With ACR50 as the endpoint. C. With ACR70 as the endpoint. D. The analysis of adverse events. Figure S6: Inconsistent assessment. A. With ACR20 as the endpoint. B. With ACR50 as the endpoint. C. With ACR70 as the endpoint. D. The analysis of adverse events. Figure S7: The publication bias. A. With ACR20 as the endpoint. B. With ACR50 as the endpoint. C. With ACR70 as the endpoint. D. The analysis of adverse events. Table S1: Inverted triangle table based on ACR50. Table S2: Inverted triangle table based on ACR70. Table S3: Inverted triangle table based on adverse events. Table S4: Search strategy. [file 3181427.f1.zip › 3181427.f1/Figure S4B.pdf]

Cumulative Probabilities

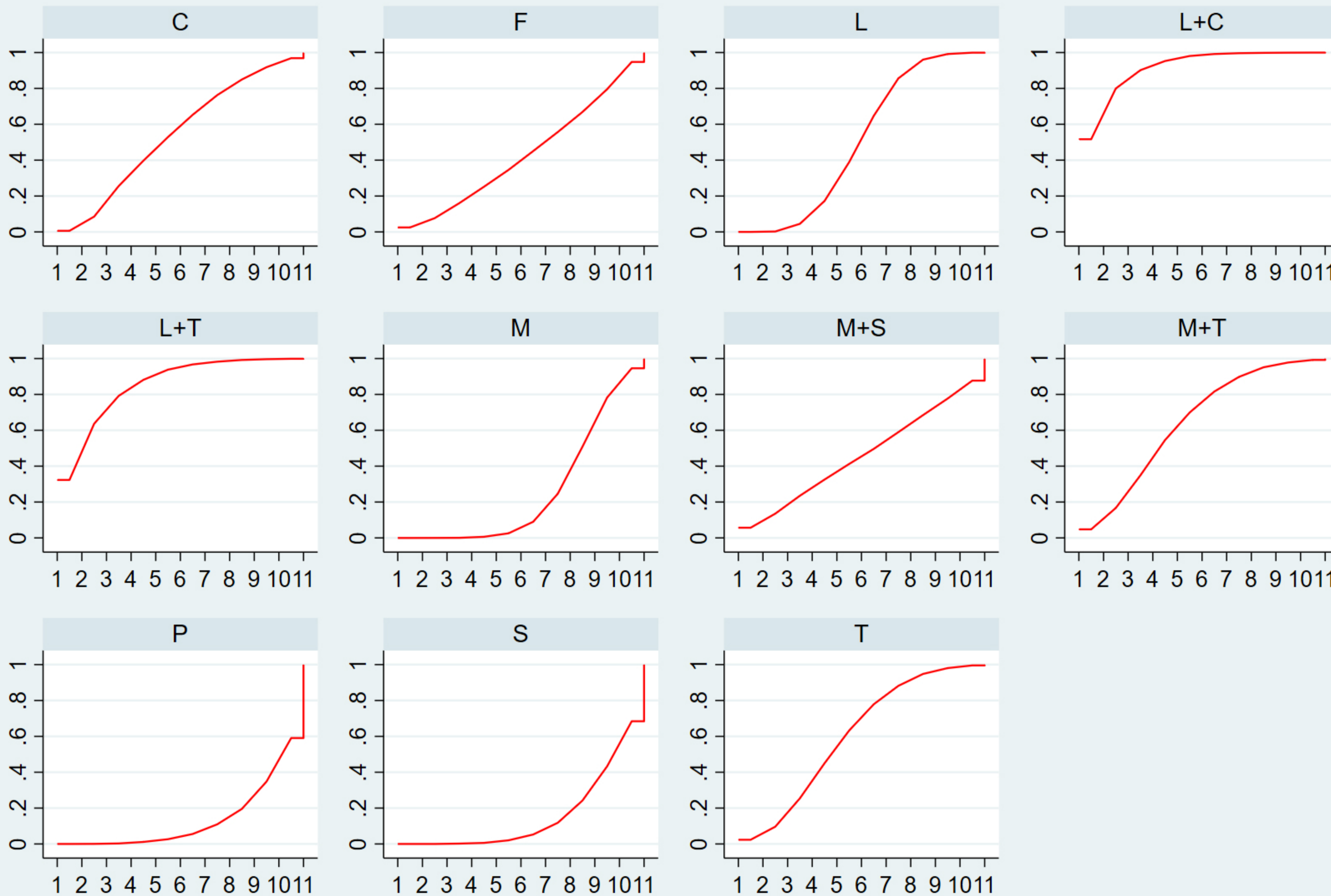

Rank

Graphs by Treatment

Supplement: Supplementary Materials — Figure S1: PRISMA-2009-Flow-Diagram-MS-Word: PRISMA flowchart. Figure S2: Risk of bias graph. Figure S3: Risk of bias summary. Figure S4: The cumulative probability diagram. A. With ACR20 as the endpoint. B. With ACR50 as the endpoint. C. With ACR70 as the endpoint. D. The analysis of adverse events. Figure S5: Forest plots. A. With ACR20 as the endpoint. B. With ACR50 as the endpoint. C. With ACR70 as the endpoint. D. The analysis of adverse events. Figure S6: Inconsistent assessment. A. With ACR20 as the endpoint. B. With ACR50 as the endpoint. C. With ACR70 as the endpoint. D. The analysis of adverse events. Figure S7: The publication bias. A. With ACR20 as the endpoint. B. With ACR50 as the endpoint. C. With ACR70 as the endpoint. D. The analysis of adverse events. Table S1: Inverted triangle table based on ACR50. Table S2: Inverted triangle table based on ACR70. Table S3: Inverted triangle table based on adverse events. Table S4: Search strategy. [file 3181427.f1.zip › 3181427.f1/Figure S4C.pdf]

Cumulative Probabilities

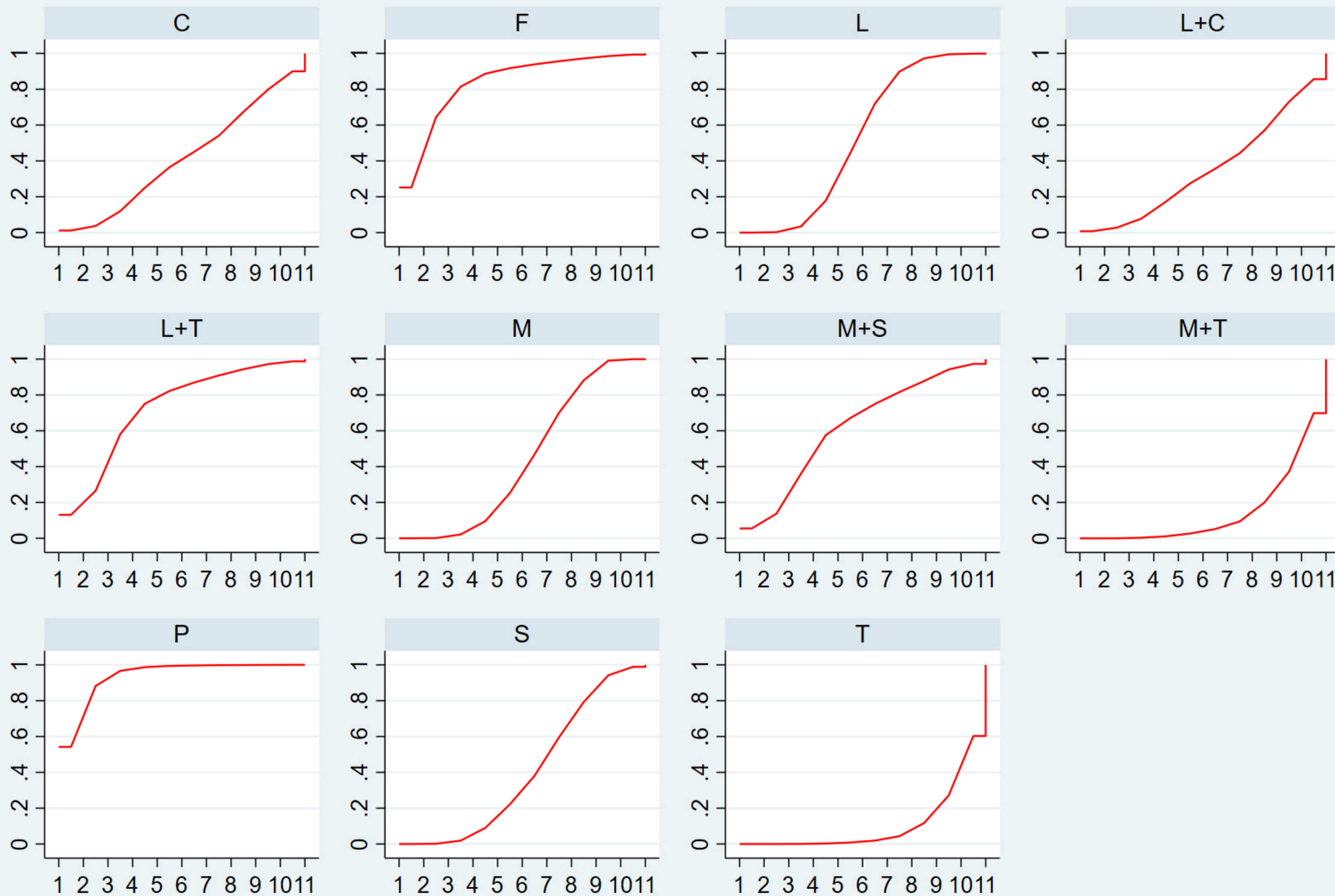

Rank

Graphs by Treatment

Supplement: Supplementary Materials — Figure S1: PRISMA-2009-Flow-Diagram-MS-Word: PRISMA flowchart. Figure S2: Risk of bias graph. Figure S3: Risk of bias summary. Figure S4: The cumulative probability diagram. A. With ACR20 as the endpoint. B. With ACR50 as the endpoint. C. With ACR70 as the endpoint. D. The analysis of adverse events. Figure S5: Forest plots. A. With ACR20 as the endpoint. B. With ACR50 as the endpoint. C. With ACR70 as the endpoint. D. The analysis of adverse events. Figure S6: Inconsistent assessment. A. With ACR20 as the endpoint. B. With ACR50 as the endpoint. C. With ACR70 as the endpoint. D. The analysis of adverse events. Figure S7: The publication bias. A. With ACR20 as the endpoint. B. With ACR50 as the endpoint. C. With ACR70 as the endpoint. D. The analysis of adverse events. Table S1: Inverted triangle table based on ACR50. Table S2: Inverted triangle table based on ACR70. Table S3: Inverted triangle table based on adverse events. Table S4: Search strategy. [file 3181427.f1.zip › 3181427.f1/Figure S4D.pdf]

# Treatment Effect

# Mean with 95%CI and 95%PrI

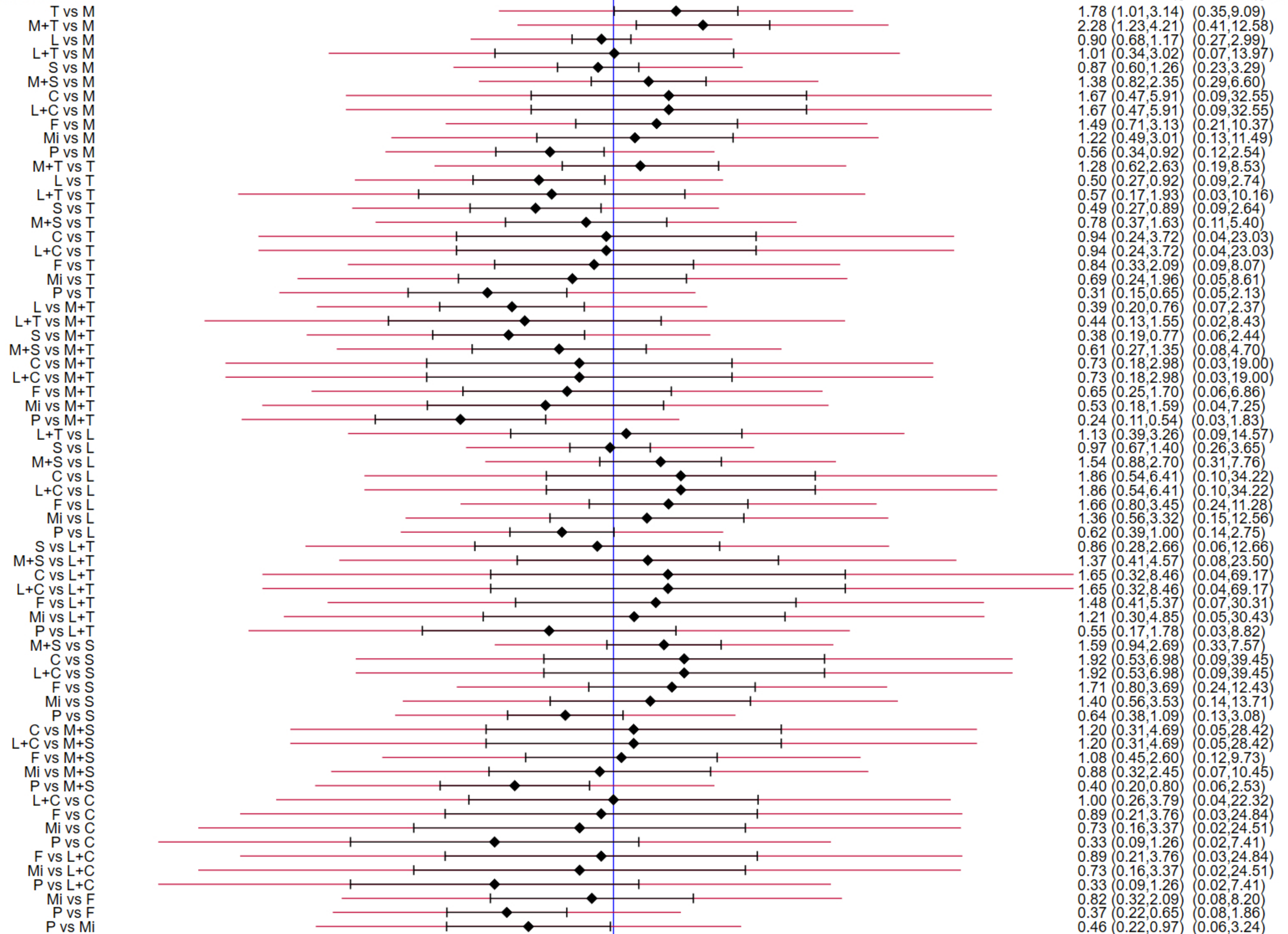

0

.1

1

8.2

67

Supplement: Supplementary Materials — Figure S1: PRISMA-2009-Flow-Diagram-MS-Word: PRISMA flowchart. Figure S2: Risk of bias graph. Figure S3: Risk of bias summary. Figure S4: The cumulative probability diagram. A. With ACR20 as the endpoint. B. With ACR50 as the endpoint. C. With ACR70 as the endpoint. D. The analysis of adverse events. Figure S5: Forest plots. A. With ACR20 as the endpoint. B. With ACR50 as the endpoint. C. With ACR70 as the endpoint. D. The analysis of adverse events. Figure S6: Inconsistent assessment. A. With ACR20 as the endpoint. B. With ACR50 as the endpoint. C. With ACR70 as the endpoint. D. The analysis of adverse events. Figure S7: The publication bias. A. With ACR20 as the endpoint. B. With ACR50 as the endpoint. C. With ACR70 as the endpoint. D. The analysis of adverse events. Table S1: Inverted triangle table based on ACR50. Table S2: Inverted triangle table based on ACR70. Table S3: Inverted triangle table based on adverse events. Table S4: Search strategy. [file 3181427.f1.zip › 3181427.f1/Figure S5A.pdf]

# Treatment Effect

# Mean with 95%CI and 95%PrI

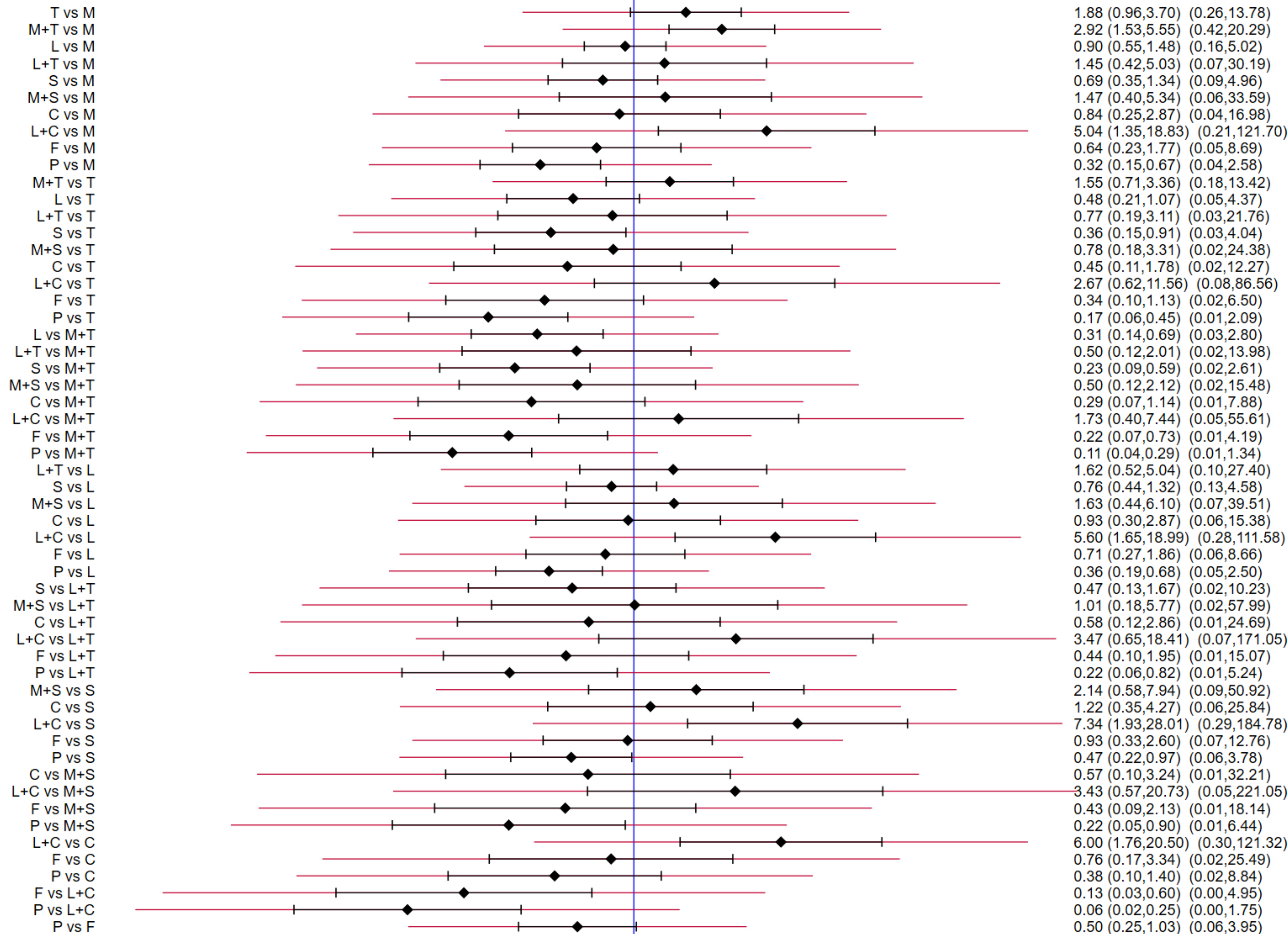

0

0

1

12

221

Supplement: Supplementary Materials — Figure S1: PRISMA-2009-Flow-Diagram-MS-Word: PRISMA flowchart. Figure S2: Risk of bias graph. Figure S3: Risk of bias summary. Figure S4: The cumulative probability diagram. A. With ACR20 as the endpoint. B. With ACR50 as the endpoint. C. With ACR70 as the endpoint. D. The analysis of adverse events. Figure S5: Forest plots. A. With ACR20 as the endpoint. B. With ACR50 as the endpoint. C. With ACR70 as the endpoint. D. The analysis of adverse events. Figure S6: Inconsistent assessment. A. With ACR20 as the endpoint. B. With ACR50 as the endpoint. C. With ACR70 as the endpoint. D. The analysis of adverse events. Figure S7: The publication bias. A. With ACR20 as the endpoint. B. With ACR50 as the endpoint. C. With ACR70 as the endpoint. D. The analysis of adverse events. Table S1: Inverted triangle table based on ACR50. Table S2: Inverted triangle table based on ACR70. Table S3: Inverted triangle table based on adverse events. Table S4: Search strategy. [file 3181427.f1.zip › 3181427.f1/Figure S5B.pdf]

# Treatment Effect

# Mean with 95%CI and 95%PrI

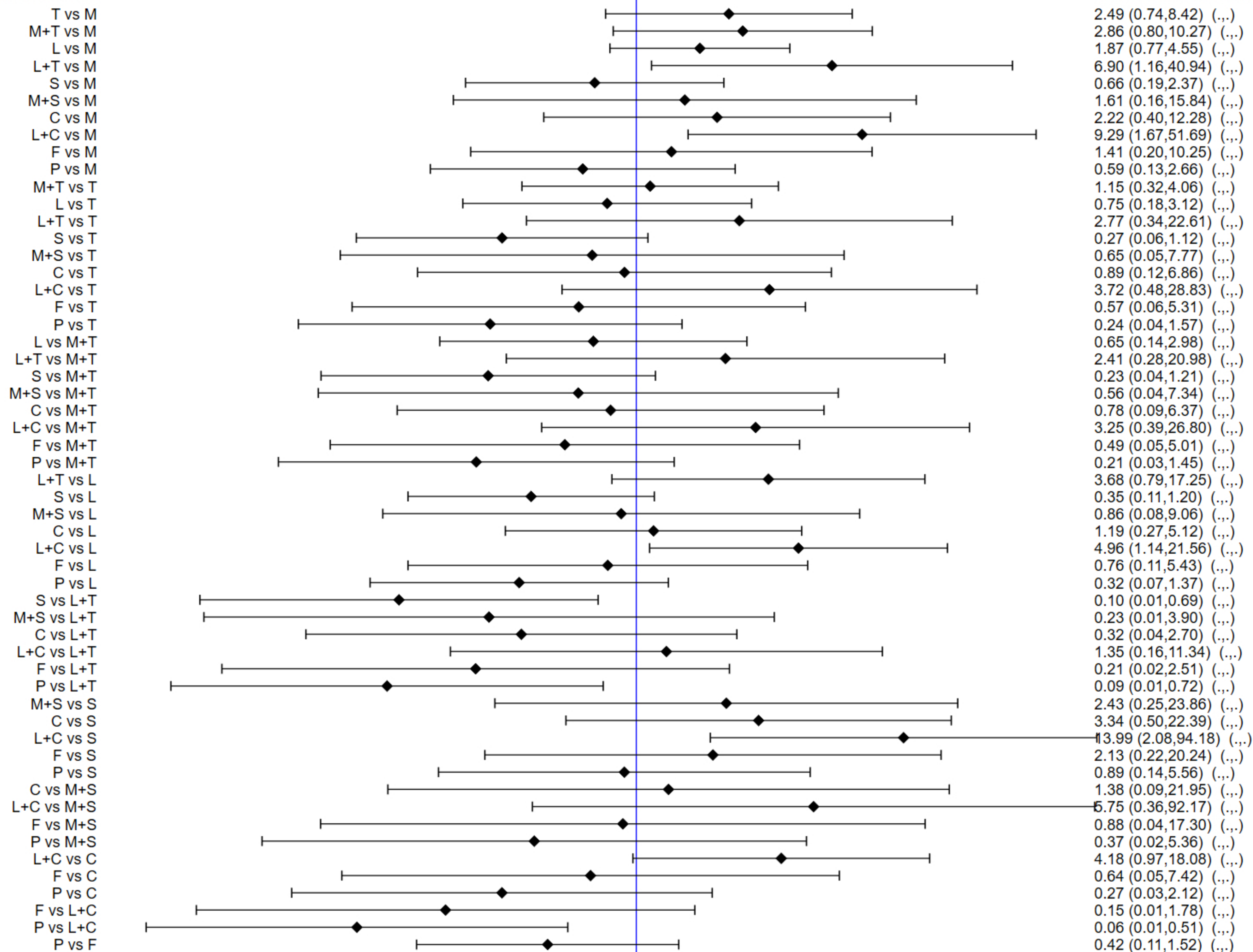

Supplement: Supplementary Materials — Figure S1: PRISMA-2009-Flow-Diagram-MS-Word: PRISMA flowchart. Figure S2: Risk of bias graph. Figure S3: Risk of bias summary. Figure S4: The cumulative probability diagram. A. With ACR20 as the endpoint. B. With ACR50 as the endpoint. C. With ACR70 as the endpoint. D. The analysis of adverse events. Figure S5: Forest plots. A. With ACR20 as the endpoint. B. With ACR50 as the endpoint. C. With ACR70 as the endpoint. D. The analysis of adverse events. Figure S6: Inconsistent assessment. A. With ACR20 as the endpoint. B. With ACR50 as the endpoint. C. With ACR70 as the endpoint. D. The analysis of adverse events. Figure S7: The publication bias. A. With ACR20 as the endpoint. B. With ACR50 as the endpoint. C. With ACR70 as the endpoint. D. The analysis of adverse events. Table S1: Inverted triangle table based on ACR50. Table S2: Inverted triangle table based on ACR70. Table S3: Inverted triangle table based on adverse events. Table S4: Search strategy. [file 3181427.f1.zip › 3181427.f1/Figure S5C.pdf]

# Treatment Effect

# Mean with 95%CI and 95%PrI

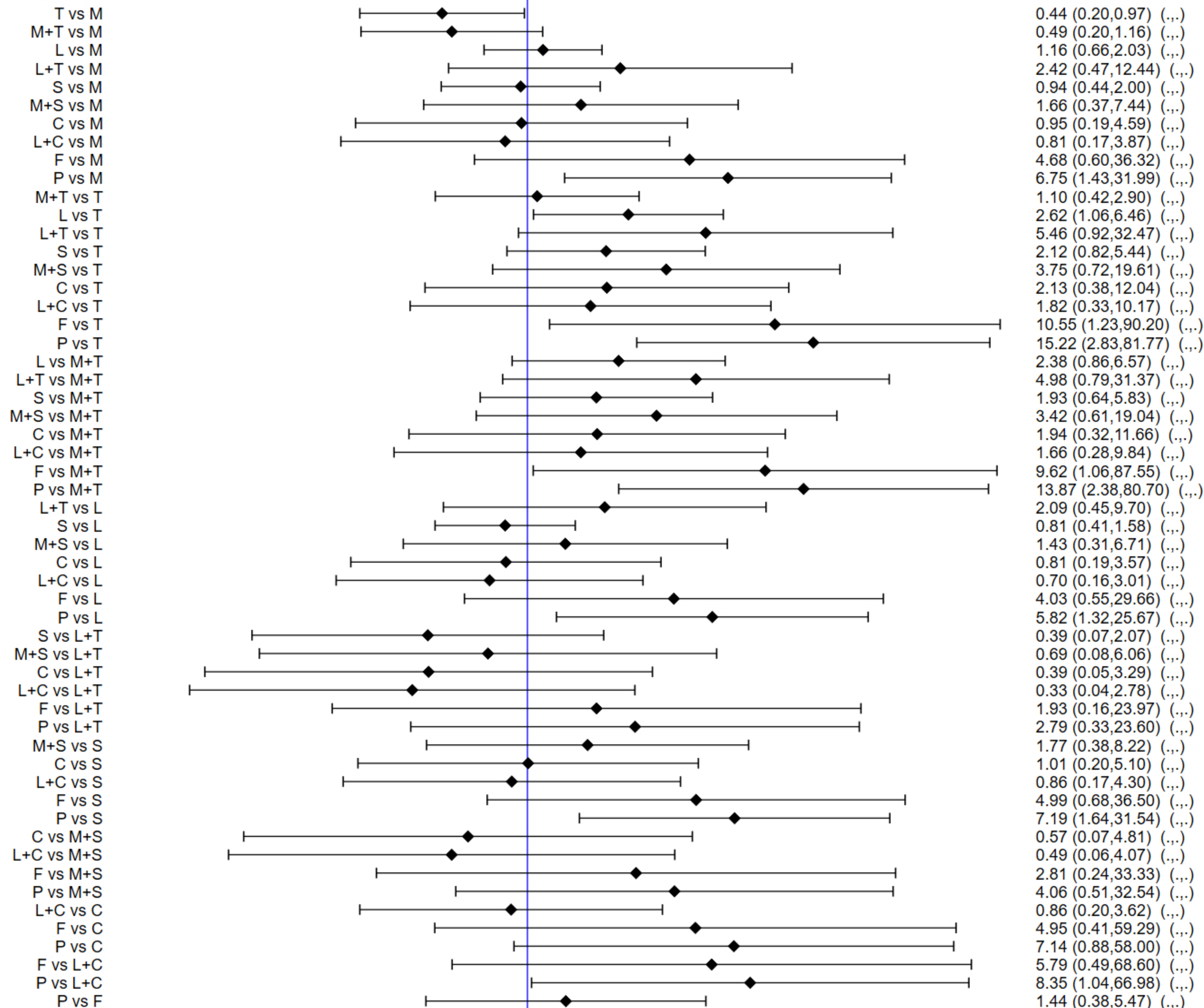

0

.3

1

14

90

Supplement: Supplementary Materials — Figure S1: PRISMA-2009-Flow-Diagram-MS-Word: PRISMA flowchart. Figure S2: Risk of bias graph. Figure S3: Risk of bias summary. Figure S4: The cumulative probability diagram. A. With ACR20 as the endpoint. B. With ACR50 as the endpoint. C. With ACR70 as the endpoint. D. The analysis of adverse events. Figure S5: Forest plots. A. With ACR20 as the endpoint. B. With ACR50 as the endpoint. C. With ACR70 as the endpoint. D. The analysis of adverse events. Figure S6: Inconsistent assessment. A. With ACR20 as the endpoint. B. With ACR50 as the endpoint. C. With ACR70 as the endpoint. D. The analysis of adverse events. Figure S7: The publication bias. A. With ACR20 as the endpoint. B. With ACR50 as the endpoint. C. With ACR70 as the endpoint. D. The analysis of adverse events. Table S1: Inverted triangle table based on ACR50. Table S2: Inverted triangle table based on ACR70. Table S3: Inverted triangle table based on adverse events. Table S4: Search strategy. [file 3181427.f1.zip › 3181427.f1/Figure S5D.pdf]

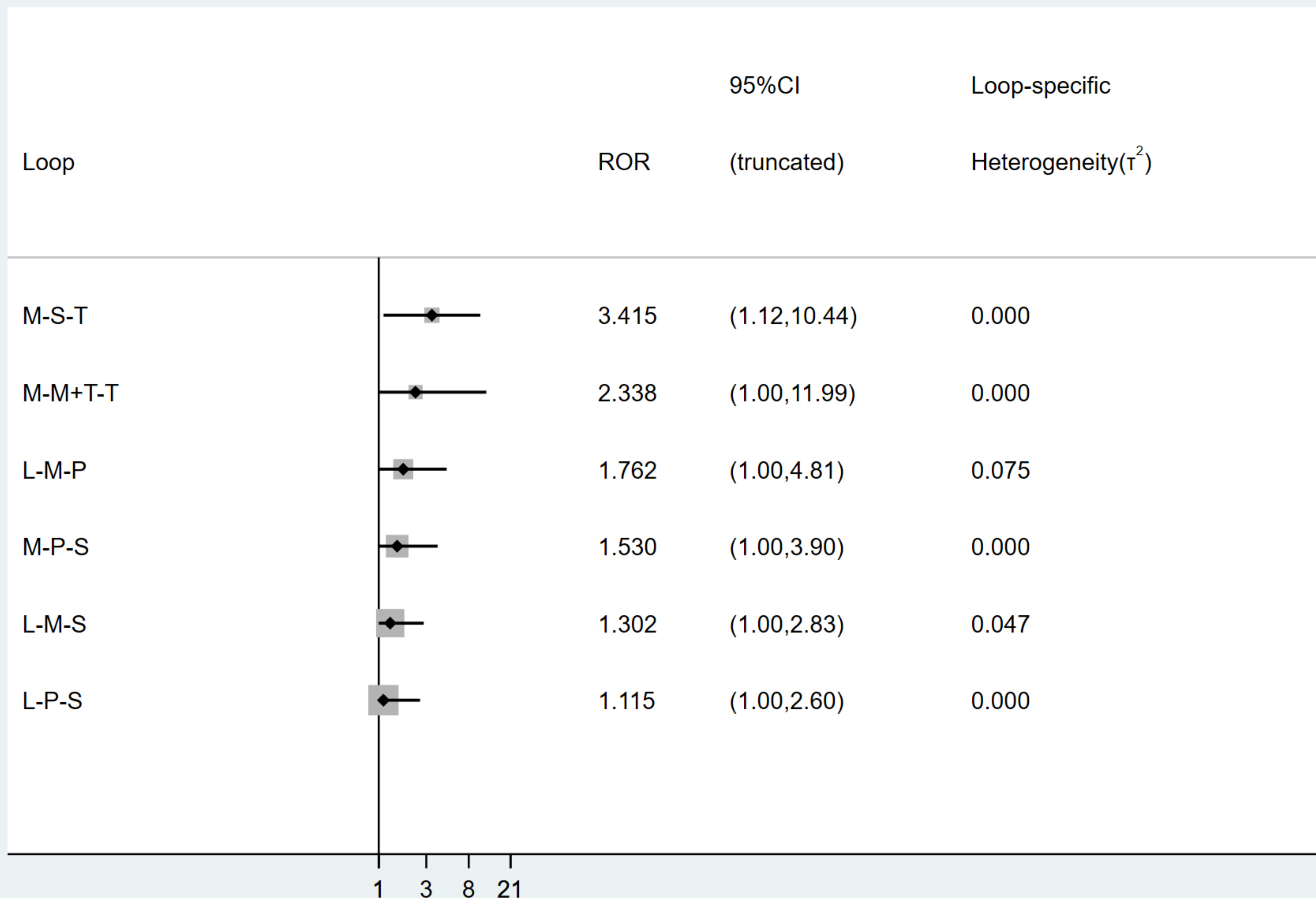

\*\*\* Loop(s) [C-L-L+C] [M-M+S-S] are formed only by multi-arm trial(s) - Consistent by definition

Supplement: Supplementary Materials — Figure S1: PRISMA-2009-Flow-Diagram-MS-Word: PRISMA flowchart. Figure S2: Risk of bias graph. Figure S3: Risk of bias summary. Figure S4: The cumulative probability diagram. A. With ACR20 as the endpoint. B. With ACR50 as the endpoint. C. With ACR70 as the endpoint. D. The analysis of adverse events. Figure S5: Forest plots. A. With ACR20 as the endpoint. B. With ACR50 as the endpoint. C. With ACR70 as the endpoint. D. The analysis of adverse events. Figure S6: Inconsistent assessment. A. With ACR20 as the endpoint. B. With ACR50 as the endpoint. C. With ACR70 as the endpoint. D. The analysis of adverse events. Figure S7: The publication bias. A. With ACR20 as the endpoint. B. With ACR50 as the endpoint. C. With ACR70 as the endpoint. D. The analysis of adverse events. Table S1: Inverted triangle table based on ACR50. Table S2: Inverted triangle table based on ACR70. Table S3: Inverted triangle table based on adverse events. Table S4: Search strategy. [file 3181427.f1.zip › 3181427.f1/Figure S6A.pdf]

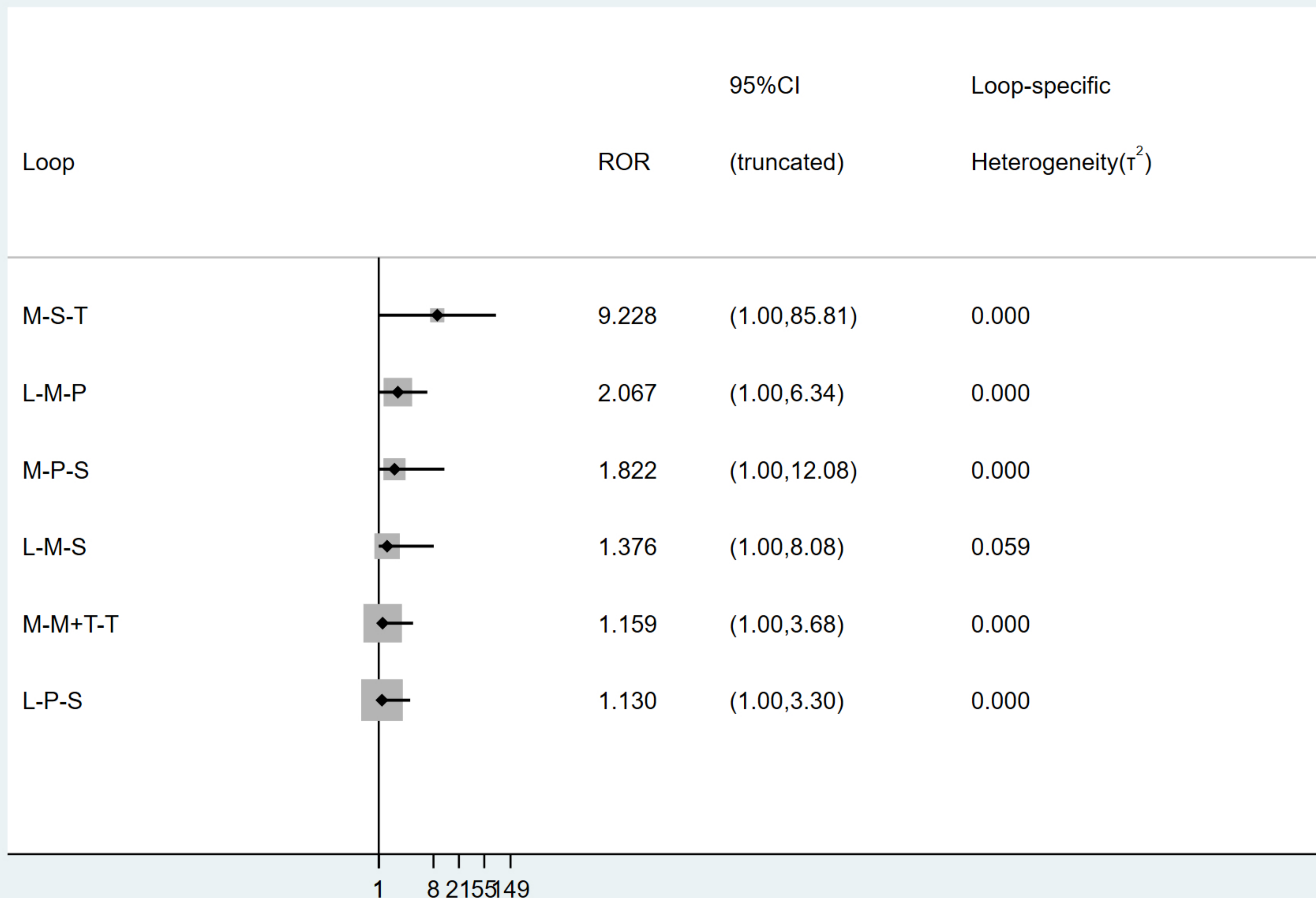

\*\*\* Loop(s) [C-C+L-L] [M-S-M+S] are formed only by multi-arm trial(s) - Consistent by definition

Supplement: Supplementary Materials — Figure S1: PRISMA-2009-Flow-Diagram-MS-Word: PRISMA flowchart. Figure S2: Risk of bias graph. Figure S3: Risk of bias summary. Figure S4: The cumulative probability diagram. A. With ACR20 as the endpoint. B. With ACR50 as the endpoint. C. With ACR70 as the endpoint. D. The analysis of adverse events. Figure S5: Forest plots. A. With ACR20 as the endpoint. B. With ACR50 as the endpoint. C. With ACR70 as the endpoint. D. The analysis of adverse events. Figure S6: Inconsistent assessment. A. With ACR20 as the endpoint. B. With ACR50 as the endpoint. C. With ACR70 as the endpoint. D. The analysis of adverse events. Figure S7: The publication bias. A. With ACR20 as the endpoint. B. With ACR50 as the endpoint. C. With ACR70 as the endpoint. D. The analysis of adverse events. Table S1: Inverted triangle table based on ACR50. Table S2: Inverted triangle table based on ACR70. Table S3: Inverted triangle table based on adverse events. Table S4: Search strategy. [file 3181427.f1.zip › 3181427.f1/Figure S6B.pdf]

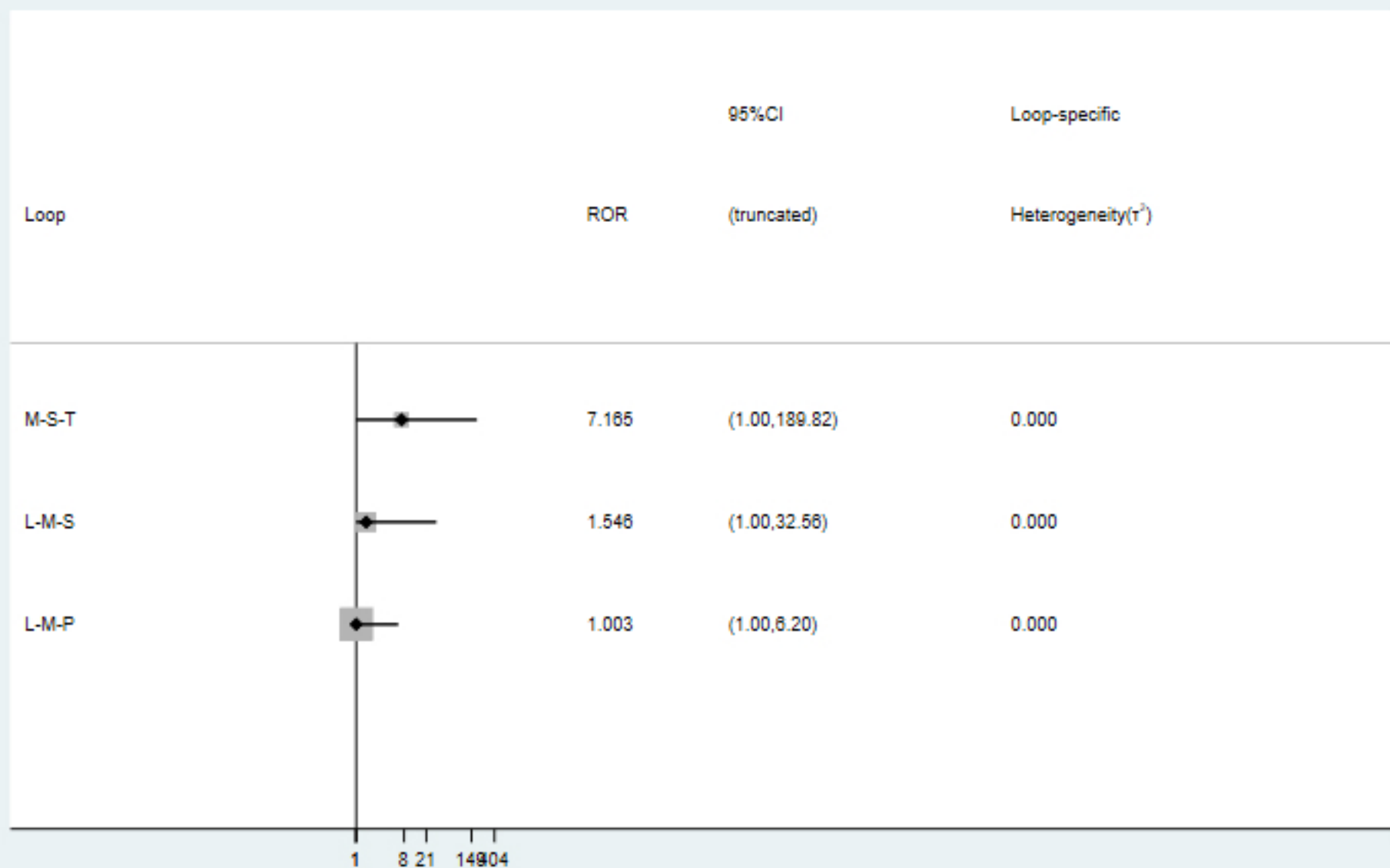

\*\*\* Loop(s) [C-L-L+C] [M-M+S-S] [M-M+T-T] are formed only by multi-arm trial(s) - Consistent by definition

Supplement: Supplementary Materials — Figure S1: PRISMA-2009-Flow-Diagram-MS-Word: PRISMA flowchart. Figure S2: Risk of bias graph. Figure S3: Risk of bias summary. Figure S4: The cumulative probability diagram. A. With ACR20 as the endpoint. B. With ACR50 as the endpoint. C. With ACR70 as the endpoint. D. The analysis of adverse events. Figure S5: Forest plots. A. With ACR20 as the endpoint. B. With ACR50 as the endpoint. C. With ACR70 as the endpoint. D. The analysis of adverse events. Figure S6: Inconsistent assessment. A. With ACR20 as the endpoint. B. With ACR50 as the endpoint. C. With ACR70 as the endpoint. D. The analysis of adverse events. Figure S7: The publication bias. A. With ACR20 as the endpoint. B. With ACR50 as the endpoint. C. With ACR70 as the endpoint. D. The analysis of adverse events. Table S1: Inverted triangle table based on ACR50. Table S2: Inverted triangle table based on ACR70. Table S3: Inverted triangle table based on adverse events. Table S4: Search strategy. [file 3181427.f1.zip › 3181427.f1/Figure S6C.pdf]

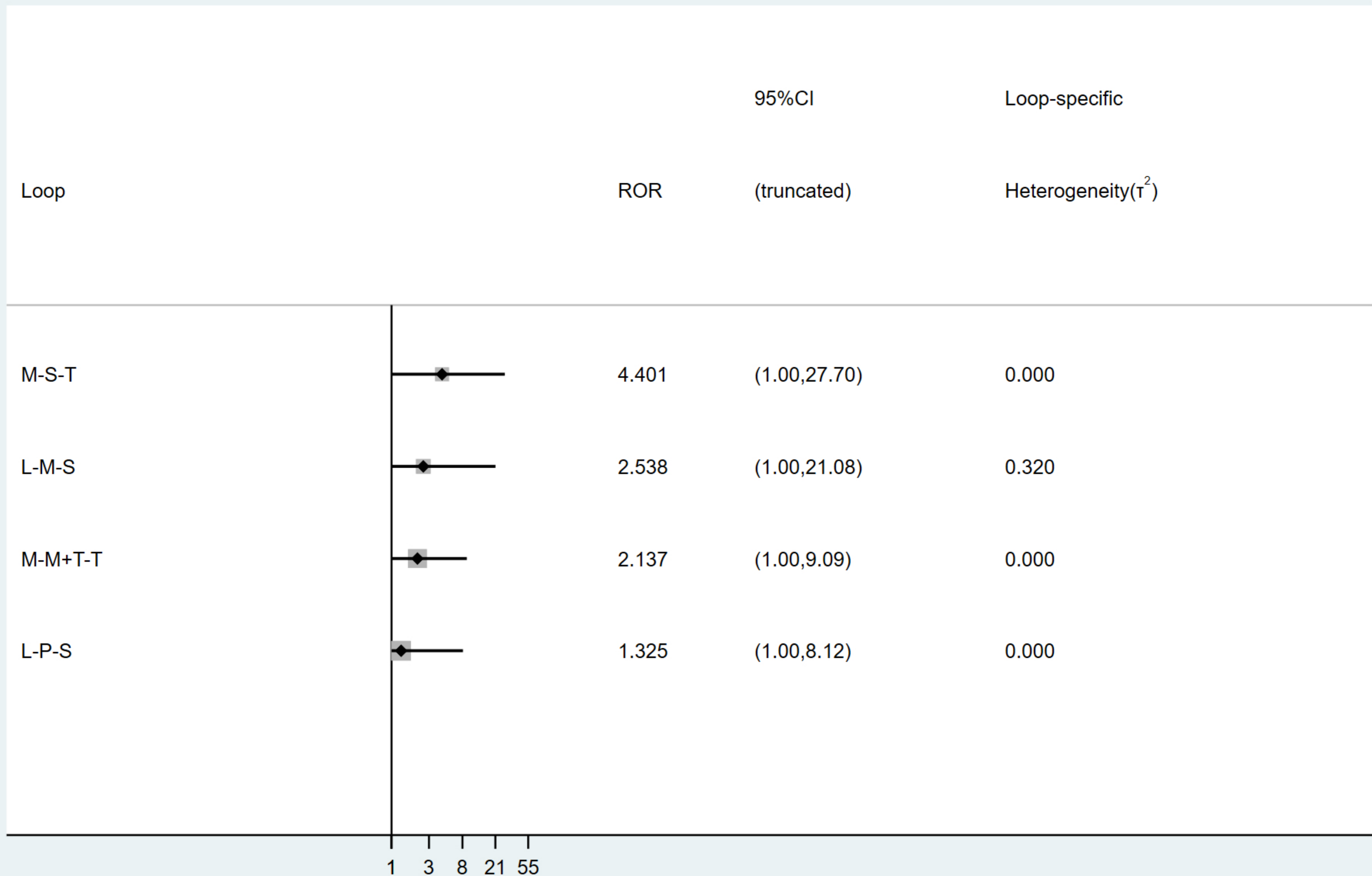

\*\*\* Loop(s) [C-L-L+C] [M-M+S-S] are formed only by multi-arm trial(s) - Consistent by definition

Supplement: Supplementary Materials — Figure S1: PRISMA-2009-Flow-Diagram-MS-Word: PRISMA flowchart. Figure S2: Risk of bias graph. Figure S3: Risk of bias summary. Figure S4: The cumulative probability diagram. A. With ACR20 as the endpoint. B. With ACR50 as the endpoint. C. With ACR70 as the endpoint. D. The analysis of adverse events. Figure S5: Forest plots. A. With ACR20 as the endpoint. B. With ACR50 as the endpoint. C. With ACR70 as the endpoint. D. The analysis of adverse events. Figure S6: Inconsistent assessment. A. With ACR20 as the endpoint. B. With ACR50 as the endpoint. C. With ACR70 as the endpoint. D. The analysis of adverse events. Figure S7: The publication bias. A. With ACR20 as the endpoint. B. With ACR50 as the endpoint. C. With ACR70 as the endpoint. D. The analysis of adverse events. Table S1: Inverted triangle table based on ACR50. Table S2: Inverted triangle table based on ACR70. Table S3: Inverted triangle table based on adverse events. Table S4: Search strategy. [file 3181427.f1.zip › 3181427.f1/Figure S6D.pdf]

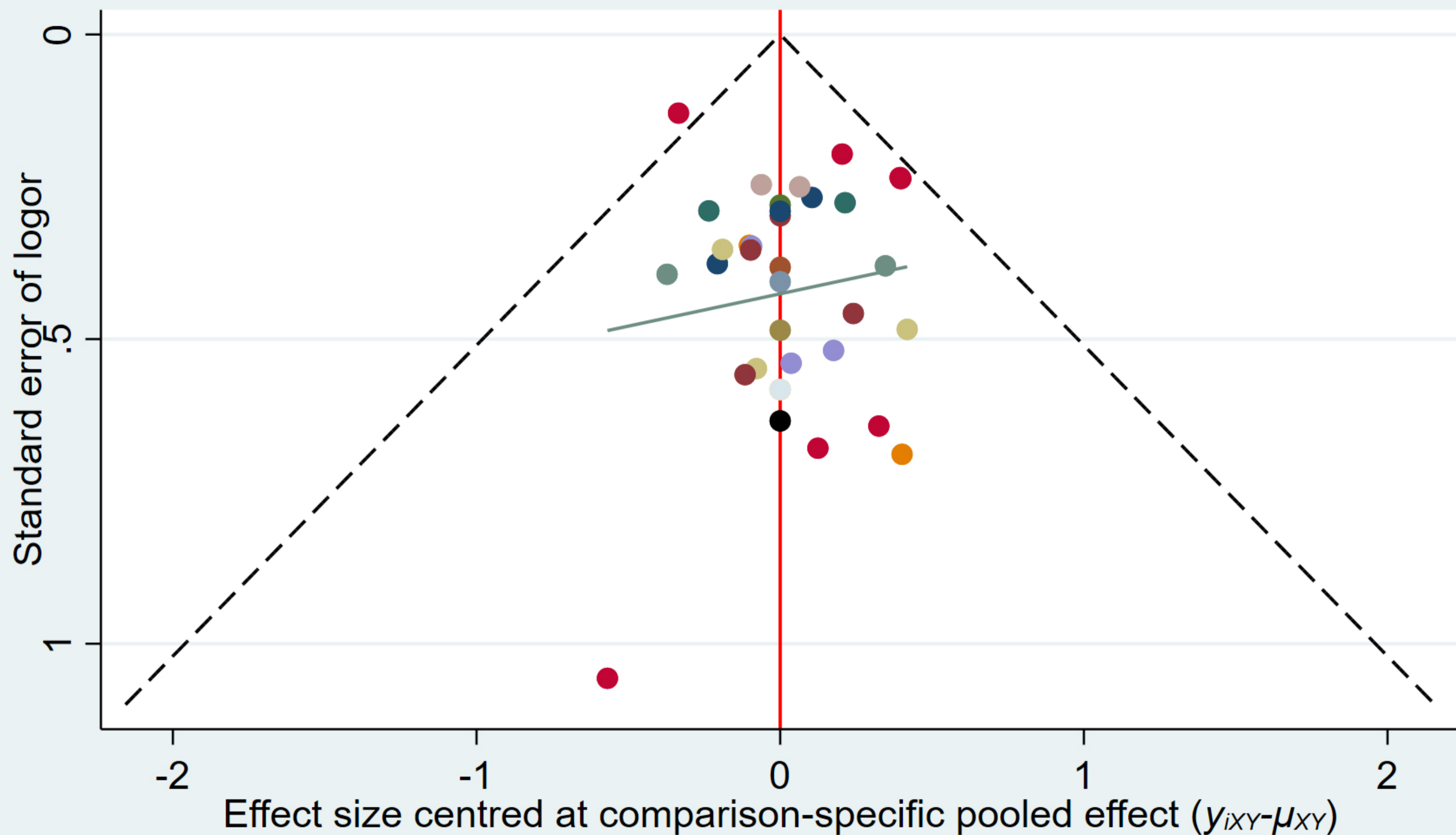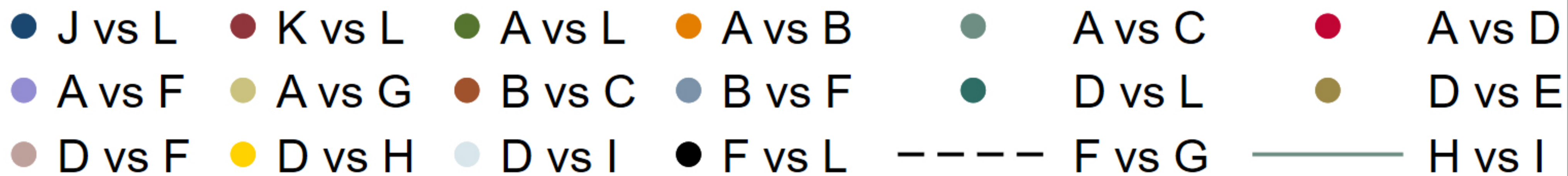

Supplement: Supplementary Materials — Figure S1: PRISMA-2009-Flow-Diagram-MS-Word: PRISMA flowchart. Figure S2: Risk of bias graph. Figure S3: Risk of bias summary. Figure S4: The cumulative probability diagram. A. With ACR20 as the endpoint. B. With ACR50 as the endpoint. C. With ACR70 as the endpoint. D. The analysis of adverse events. Figure S5: Forest plots. A. With ACR20 as the endpoint. B. With ACR50 as the endpoint. C. With ACR70 as the endpoint. D. The analysis of adverse events. Figure S6: Inconsistent assessment. A. With ACR20 as the endpoint. B. With ACR50 as the endpoint. C. With ACR70 as the endpoint. D. The analysis of adverse events. Figure S7: The publication bias. A. With ACR20 as the endpoint. B. With ACR50 as the endpoint. C. With ACR70 as the endpoint. D. The analysis of adverse events. Table S1: Inverted triangle table based on ACR50. Table S2: Inverted triangle table based on ACR70. Table S3: Inverted triangle table based on adverse events. Table S4: Search strategy. [file 3181427.f1.zip › 3181427.f1/Figure S7A.pdf]

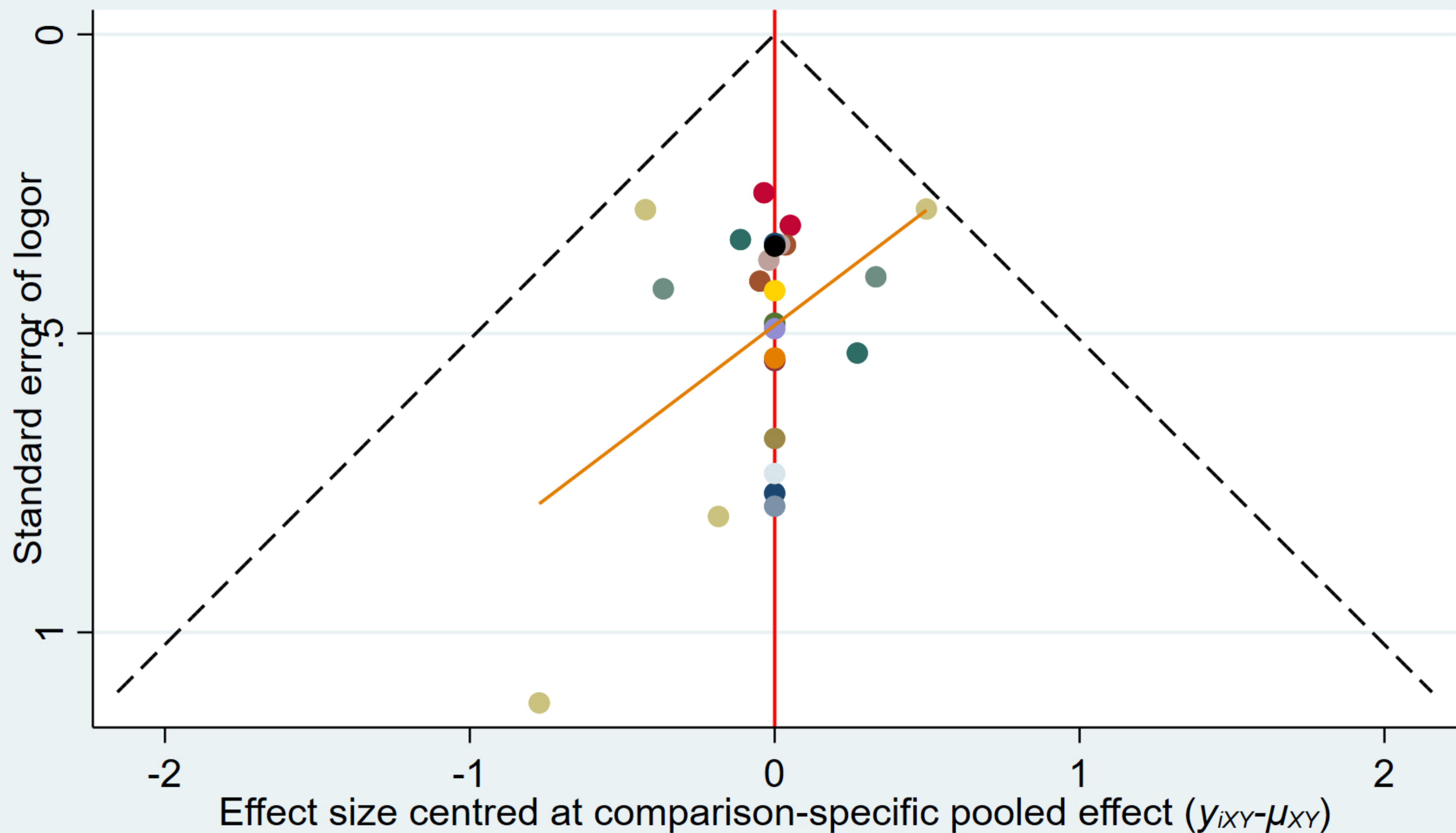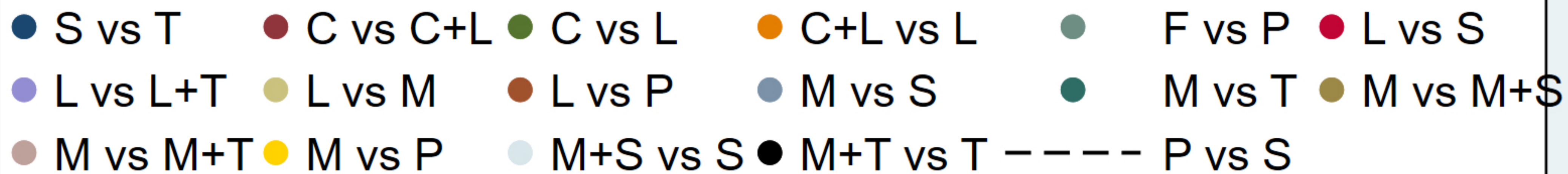

Supplement: Supplementary Materials — Figure S1: PRISMA-2009-Flow-Diagram-MS-Word: PRISMA flowchart. Figure S2: Risk of bias graph. Figure S3: Risk of bias summary. Figure S4: The cumulative probability diagram. A. With ACR20 as the endpoint. B. With ACR50 as the endpoint. C. With ACR70 as the endpoint. D. The analysis of adverse events. Figure S5: Forest plots. A. With ACR20 as the endpoint. B. With ACR50 as the endpoint. C. With ACR70 as the endpoint. D. The analysis of adverse events. Figure S6: Inconsistent assessment. A. With ACR20 as the endpoint. B. With ACR50 as the endpoint. C. With ACR70 as the endpoint. D. The analysis of adverse events. Figure S7: The publication bias. A. With ACR20 as the endpoint. B. With ACR50 as the endpoint. C. With ACR70 as the endpoint. D. The analysis of adverse events. Table S1: Inverted triangle table based on ACR50. Table S2: Inverted triangle table based on ACR70. Table S3: Inverted triangle table based on adverse events. Table S4: Search strategy. [file 3181427.f1.zip › 3181427.f1/Figure S7B.pdf]

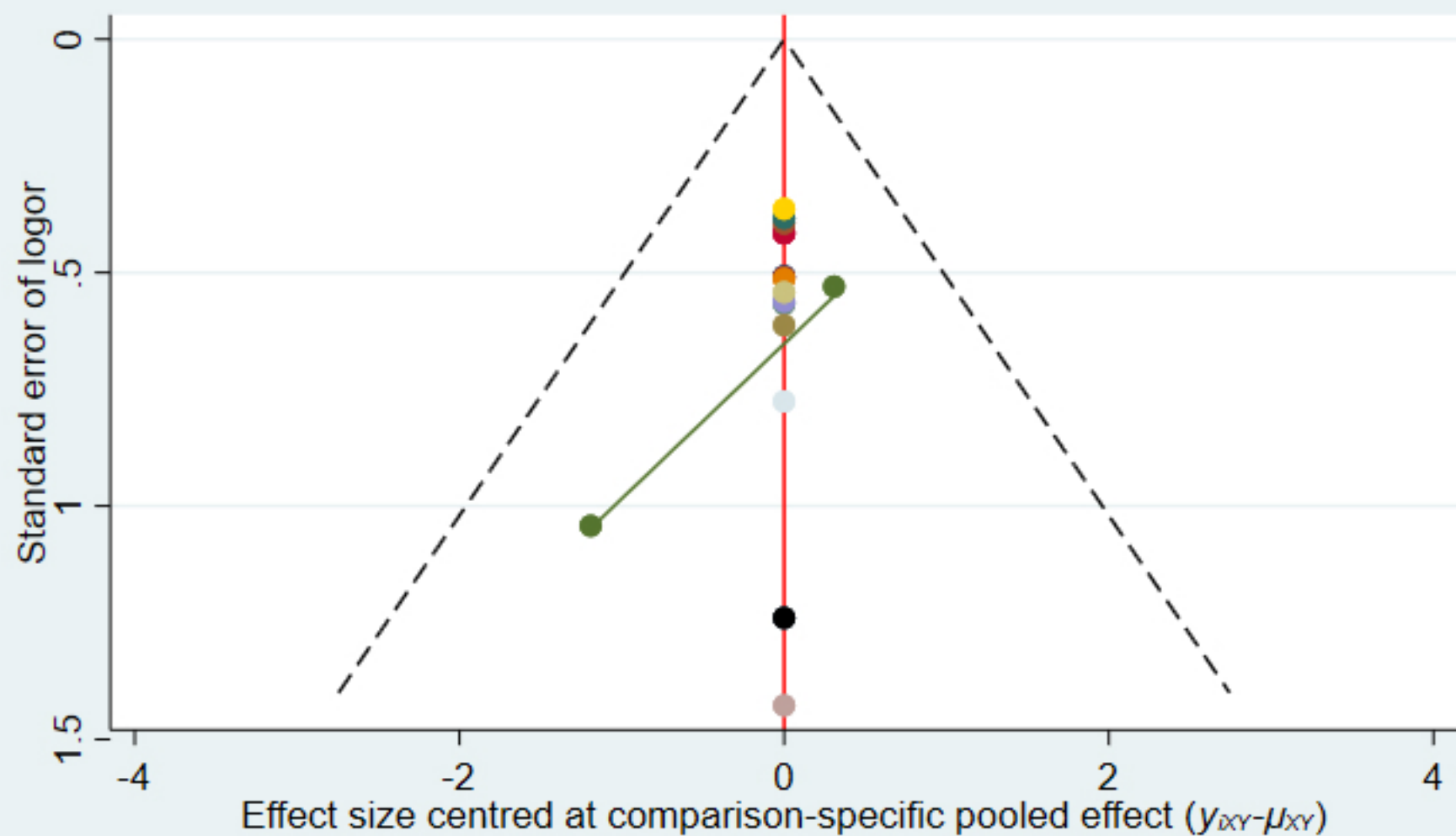

- |          |            |          |            |            |          |
|----------|------------|----------|------------|------------|----------|
| ● C vs L | ● C vs L+C | ● F vs P | ● L vs L+C | ● L vs L+T | ● L vs M |
| ● L vs P | ● L vs S   | ● M vs T | ● M vs M+S | ● M vs M+T | ● M vs P |
| ● M vs S | ● M+T vs T | ● S vs T | ● S vs M+S |            |          |

Supplement: Supplementary Materials — Figure S1: PRISMA-2009-Flow-Diagram-MS-Word: PRISMA flowchart. Figure S2: Risk of bias graph. Figure S3: Risk of bias summary. Figure S4: The cumulative probability diagram. A. With ACR20 as the endpoint. B. With ACR50 as the endpoint. C. With ACR70 as the endpoint. D. The analysis of adverse events. Figure S5: Forest plots. A. With ACR20 as the endpoint. B. With ACR50 as the endpoint. C. With ACR70 as the endpoint. D. The analysis of adverse events. Figure S6: Inconsistent assessment. A. With ACR20 as the endpoint. B. With ACR50 as the endpoint. C. With ACR70 as the endpoint. D. The analysis of adverse events. Figure S7: The publication bias. A. With ACR20 as the endpoint. B. With ACR50 as the endpoint. C. With ACR70 as the endpoint. D. The analysis of adverse events. Table S1: Inverted triangle table based on ACR50. Table S2: Inverted triangle table based on ACR70. Table S3: Inverted triangle table based on adverse events. Table S4: Search strategy. [file 3181427.f1.zip › 3181427.f1/Figure S7C.pdf]

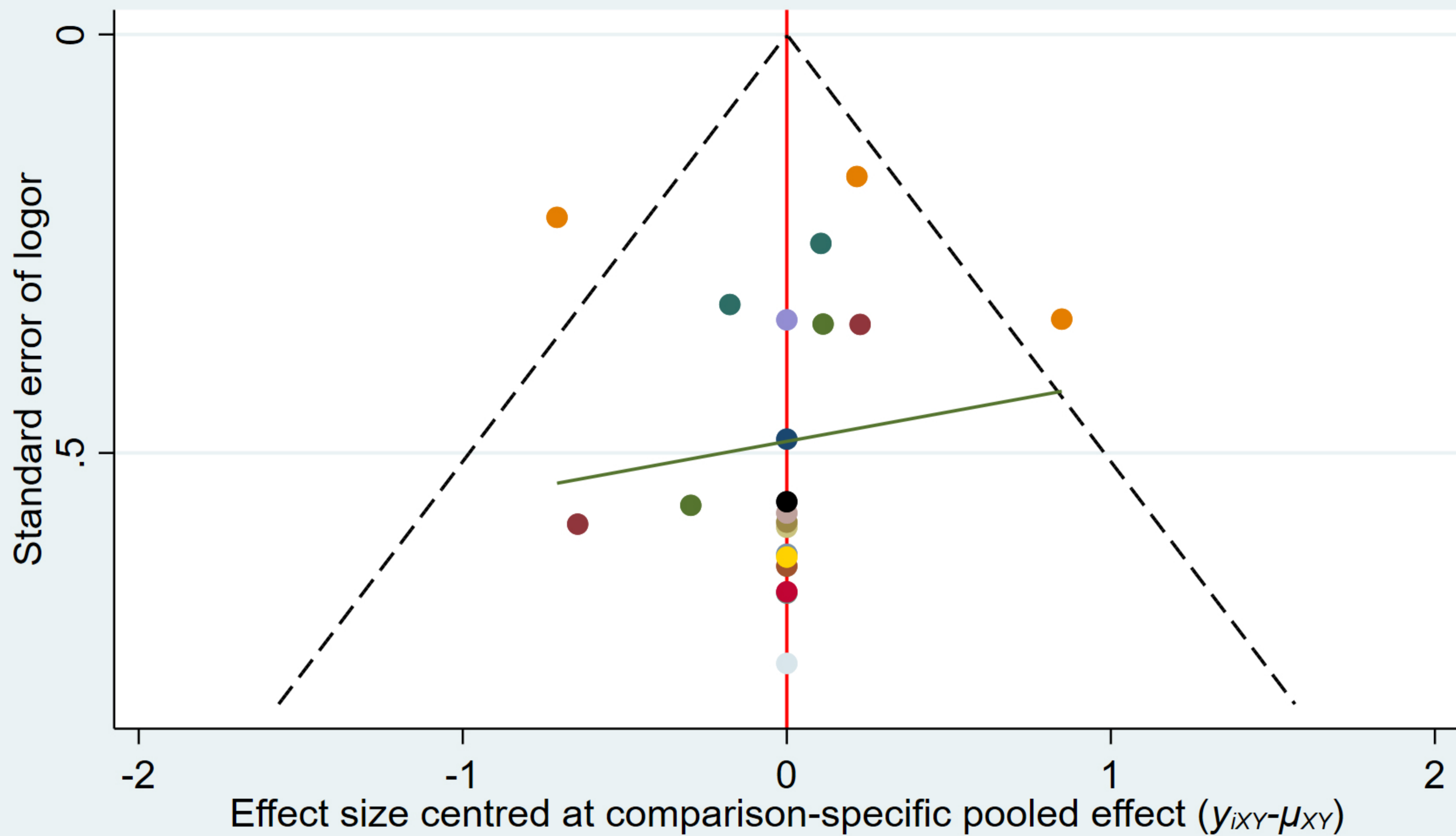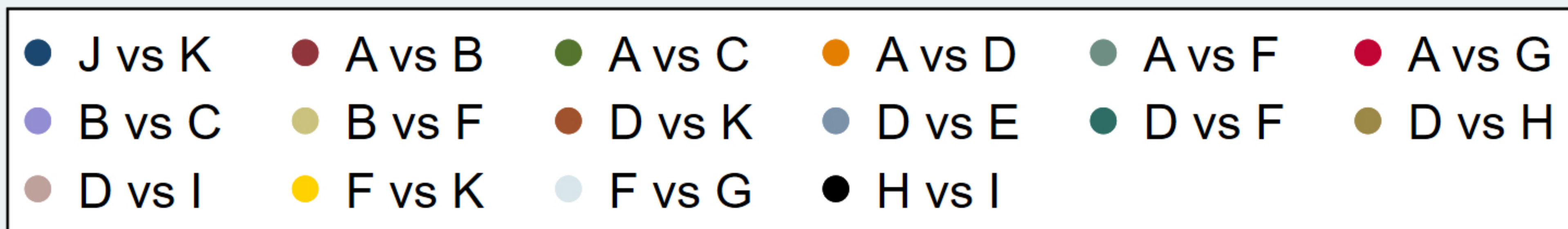

Supplement: Supplementary Materials — Figure S1: PRISMA-2009-Flow-Diagram-MS-Word: PRISMA flowchart. Figure S2: Risk of bias graph. Figure S3: Risk of bias summary. Figure S4: The cumulative probability diagram. A. With ACR20 as the endpoint. B. With ACR50 as the endpoint. C. With ACR70 as the endpoint. D. The analysis of adverse events. Figure S5: Forest plots. A. With ACR20 as the endpoint. B. With ACR50 as the endpoint. C. With ACR70 as the endpoint. D. The analysis of adverse events. Figure S6: Inconsistent assessment. A. With ACR20 as the endpoint. B. With ACR50 as the endpoint. C. With ACR70 as the endpoint. D. The analysis of adverse events. Figure S7: The publication bias. A. With ACR20 as the endpoint. B. With ACR50 as the endpoint. C. With ACR70 as the endpoint. D. The analysis of adverse events. Table S1: Inverted triangle table based on ACR50. Table S2: Inverted triangle table based on ACR70. Table S3: Inverted triangle table based on adverse events. Table S4: Search strategy. [file 3181427.f1.zip › 3181427.f1/Figure S7D.pdf]
