# Supplementary material for: A Systematic Review and Network Meta-Analysis about the Efficacy and Safety of Tripterygium wilfordii Hook F in Rheumatoid Arthritis
Source: Evid Based Complement Alternat Med. 2022 May 10;2022:3181427. doi: 10.1155/2022/3181427 (PMC9113883; doi:10.1155/2022/3181427)
Supplement: Supplementary Materials — Figure S1: PRISMA-2009-Flow-Diagram-MS-Word: PRISMA flowchart. Figure S2: Risk of bias graph. Figure S3: Risk of bias summary. Figure S4: The cumulative probability diagram. A. With ACR20 as the endpoint. B. With ACR50 as the endpoint. C. With ACR70 as the endpoint. D. The analysis of adverse events. Figure S5: Forest plots. A. With ACR20 as the endpoint. B. With ACR50 as the endpoint. C. With ACR70 as the endpoint. D. The analysis of adverse events. Figure S6: Inconsistent assessment. A. With ACR20 as the endpoint. B. With ACR50 as the endpoint. C. With ACR70 as the endpoint. D. The analysis of adverse events. Figure S7: The publication bias. A. With ACR20 as the endpoint. B. With ACR50 as the endpoint. C. With ACR70 as the endpoint. D. The analysis of adverse events. Table S1: Inverted triangle table based on ACR50. Table S2: Inverted triangle table based on ACR70. Table S3: Inverted triangle table based on adverse events. Table S4: Search strategy. [file 3181427.f1.zip › 3181427.f1/Figure S1.PRISMA-2009-Flow-Diagram-MS-Word.doc]

**PRISMA 2009 Flow Diagram**

**Screening**

**Included**

**Eligibility**

**Identification**

Records identified through database searching
(n = 16987 )

Additional records identified through other sources
(n = 0 )

Records after duplicates removed
(n = 1936)

Records screened
(n = 15051 )

Records excluded
(n = 14938 )

Full-text articles assessed for eligibility
(n = 113 )

Full-text articles excluded, with reasons
(n = 92 )

Studies included in qualitative synthesis
(n = 21 )

Studies included in quantitative synthesis (meta-analysis)
(n = 21 )
